# CD177 Deficiency Defines a Stable Subtype of Human Neutrophil Granulocytes with Tumor Promoting Activity

**DOI:** 10.1002/advs.76236

**Published:** 2026-06-22

**Authors:** Marcel Jung, Alexander Beer, Susmita Ghosh, Ekaterina Pylaeva, Belal Alshaar, Tobias Tertel, Nils Bastian Leimkühler, Thomas Schroeder, Janine Gronewold, Nina Hagemann, Ayan Mohamud Yusuf, Benedikt Frank, Yiqiao Zhang, Dennis Nagel, Kim Schloeßer, Laura Karsch, Emily Hedtfeld, Sabrina Lohmann, Kathrin Blank, Andreas Kraus, Max Krumbein, Nastassia Kabankova, Hongxiao Wang, Almke Bader, Mathis Richter, Fengjun Zhang, Raphael Chevre, Bernd Giebel, Stephan Lang, Anika Grüneboom, Daniela Maier‐Begandt, Anja Hasenberg, Oliver Soehnlein, Hans Christian Reinhardt, Sven Heiles, Jianxu Chen, Jadwiga Jablonska, Albert Sickmann, Dirk M. Hermann, Matthias Gunzer

**Affiliations:** ^1^ Institute For Experimental Immunology and Imaging University Hospital University of Duisburg‐Essen Essen Germany; ^2^ Leibniz‐Institut Für Analytische Wissenschaften – ISAS – e.V. Dortmund Germany; ^3^ Department of Otorhinolaryngology University Hospital Essen University Duisburg‐Essen Essen Germany; ^4^ Institute For Transfusion Medicine University Hospital Essen University of Duisburg‐Essen Essen Germany; ^5^ Department of Hematology and Stem Cell Transplantation West‐German Cancer Center University Hospital Essen Essen Germany; ^6^ Department of Neurology University Hospital Essen University of Duisburg‐Essen Essen Germany; ^7^ Academy for Multidisciplinary Studies Capital Normal University Beijing China; ^8^ Institute of Cardiovascular Physiology and Pathophysiology Biomedical Center Ludwig‐Maximilians‐Universität München Munich Germany; ^9^ Institute For Experimental Pathology Centre of Molecular Biology of Inflammation University of Münster Münster Germany; ^10^ Partner Site Düsseldorf/Essen German Cancer Consortium (DKTK) Essen Germany; ^11^ West German Cancer Center University Hospital Essen Essen Germany; ^12^ Center For Molecular Biotechnology University Hospital Essen Essen Germany; ^13^ Faculty of Chemistry University of Duisburg‐Essen Essen Germany; ^14^ Medical Faculty Ruhr‐Universität Bochum Bochum Germany

**Keywords:** cancer, CD177, human neutrophils, inflammation, transmigration

## Abstract

The surface protein CD177 on human neutrophil granulocytes is linked to tissue infiltration. In most individuals, circulating neutrophils comprise distinct fractions of CD177‐expressing (CD177^+^) and non‐expressing (CD177^−^) cells, which differ functionally and influence disease outcomes. Currently, CD177 is considered a dynamic activation marker ultimately expressed by most neutrophils. Here, we show that, instead, CD177^−^‐neutrophils represent a stable subpopulation that never acquires CD177. CD177^+^‐ to CD177^−^‐neutrophil ratios persist over time in individuals, regardless of circadian rhythms or inflammation. Neutrophils remain CD177^−^ during in vitro stimulation and are morphologically similar to CD177^+^‐cells, but differ markedly regarding protein composition. Following stem cell transplantation, the host bone marrow defines the CD177‐pattern of mature neutrophils. Functionally, CD177^−^ neutrophils display pro‐tumoral properties, including elevated arginase expression and enhanced tumor‐supporting activity, are enriched in human head‐and‐neck cancer tissues, and associated with adverse clinical outcomes. Hence, CD177‐expression or ‐absence defines stable human neutrophil subtypes with distinct functions.

## Graphical abstract

Human neutrophils exist as two epigenetically imprinted subtypes defined by stable CD177 expression or absence — a ratio that persists across time, circadian rhythms, and inflammation. CD177^−^ neutrophils display a distinct molecular landscape enriched in arginase 1 and lipid metabolism markers, accumulate in head‐and‐neck tumors, and associate with adverse clinical outcomes, revealing CD177 as a functionally decisive neutrophil identity marker.

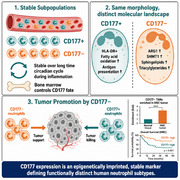

## 1 Introduction

The LU (Ly6/uPAR) domain protein family member CD177 (NB1) is a glycosylphosphatidylinositol (GPI)‐anchored membrane protein predominantly expressed on human neutrophils [1, 2]. A sizeable fraction of individuals does not express CD177 at all (CD177^null^) [3] due to pseudogene conversion [4]. However, also in the large majority of individuals expressing CD177, monoallelic silencing creates two distinct neutrophil populations, those that express CD177 (CD177^+^) and those that are CD177‐deficient (CD177^−^). Individuals show variable proportions (0‐100%) of CD177^+^ cells [5]. CD177 interacts with endothelial PECAM‐1 during transmigration [6] but CD177^−^ neutrophils were shown to also efficiently invade tissues [7]. Meanwhile, evidence is accumulating, that both neutrophil phenotypes differ functionally with high relevance for human disease. E.g., we have shown recently, that CD177^+^ cells are important for successful recovery from stroke and hence a high number of CD177^−^ cells in the circulation is associated with poor outcome [3]. In contrast, during ischemia‐reperfusion injury, CD177^+^ neutrophils appear to be problematic [8]. In addition, CD177 upregulation on circulating neutrophils has been reported in various diseases [8, 9, 10]. Importantly, the current consensus considers CD177 a dynamic marker for both, neutrophil maturation [11] and activation [12]. Hence, it is assumed that ultimately all neutrophils upregulate CD177 and become CD177^+^. However, while this notion might be true for neutrophils that already have CD177 on their surface, it has never been tested, whether initially CD177^−^ neutrophils are able to ultimately express CD177, which would be expected from a maturation marker. Here we demonstrate that, against expectations, CD177^−^ neutrophils in humans do not constitute a transitional state, but rather are a bona‐fide stable subpopulation. They cannot express CD177, since the gene is inactive and no protein is produced in these cells. While CD177 itself is mainly a useful marker to distinguish these two types of neutrophils, an in‐depth profiling of CD177^+^ and CD177^−^ neutrophils from the same hosts shows, that they are fundamentally different in molecular composition. This induces distinct functions that are relevant for disease courses, as seen here in human primary head‐and‐neck cancer.

## 2 Methods

### 2.1 Donor Cohort

All participants included within this study were aged ≥18 years. All participants were treated according to national and international guidelines and provided informed consent. Healthy volunteers were recruited locally from the investigator's personal and professional contacts, covering all sexes and diverse age‐groups.

Data on ischemic stroke patients (ISP), patients undergoing bone marrow transplantation, as well as healthy donors (HD) analyzed at Essen University Hospital were collected as part of a prospective clinical study Neutrophils: Origin, Fate & Function Stroke (NOFF‐S), supported by the German Research Foundation (DFG) funded Collaborative Research Center Trans Regio 332 (TRR332 “Neutrophils: origin, fate and function”, https://www.neutrophils.de, subproject C6) (DRKS00030825), which has been reviewed and approved by the local institutional board (21‐10271‐BO). ISPs were recruited from October 2022 onward, HDs were recruited from November 2024 onward.

Data on circadian dynamics and transmigration in healthy individuals was gained from previously generated data without the need for additional sampling (2021‐424‐f‐S).

The blood collection at LMU Munich was performed from healthy donors according to the Declaration of Helsinki and was approved by the Ethics Committee of the LMU Munich.

### 2.2 Neutrophil Isolation

Venous blood was collected in K3EDTA monovettes / EDTA Tubes. Neutrophils were isolated from whole blood using the MACSxpress Whole Blood Neutrophil Isolation Kit, human from Miltenyi Biotec (Cat. # 130‐104‐434) following manufacturer's instruction. An additional depletion of erythrocytes was included in the procedure following recommended procedure given with MACSxpress Erythrocyte Depletion Kit, human from Miltenyi Biotec (Cat. # 130‐098‐196). Protocol has been scaled according to experimental needs.

Peripheral blood samples from HNC patients were collected into 3.2% sodium citrate anticoagulant monovettes (Sarstedt; Cat. # 04.1902.001) and within 2 h after collection diluted 1:1 with PBS (Gibco; Cat. # 10010023) prior to density gradient centrifugation with Biocoll density of 1077 g/mL (Merck; Cat. # L6715). The mononuclear cell layer was discarded, and neutrophils were isolated by sedimentation over 1% polyvinyl alcohol, followed by hypotonic lysis in 0.2% NaCl solution to remove residual erythrocytes.

### 2.3 Isolation of Human T Cells

Peripheral blood was drawn into 3.8% sodium citrate anticoagulant monovettes (Sarstedt, Cat. # 02.1067.001) and mixed 1:1 with PBS (Gibco, Cat. # 14040133) before separation by density gradient centrifugation (Biocoll density 1077 g/mL, Merck, Cat. # L6115) for 30 min at 300xg (without break) at 20°C. The mononuclear cell fraction was collected, resuspended in buffer (PBS, 0.5% bovine serum albumin (R&D, Cat. # 5217), 2 mM EDTA (Sigma–Aldrich, Cat. # E9884)), and washed a total of 3 times in buffer. T cells were isolated from mononuclear cells using immunomagnetic separation (pan T cell negative selection kit, Miltenyi, Cat. # 130‐096‐535) according to the manufacturer's protocol. Isolated T cells (purity ∼95%) were resuspended in RPMIc medium (RPMI 1640 Medium (Gibco, Cat. # 11875093), 10% Fetal Bovine Serum (PAN Biotech, Cat. # P30‐3031), 1% penicillin‐streptomycin (Gibco, Cat. # 15140122), aCD3 (BioLegend, Cat. # 300402), aCD28 (BioLegend, Cat. # 302902), and rhIL‐2 (Peprotech, Cat. # 200‐02)) in concentration 1 000 000 cells/mL and co‐incubated with autologous neutrophils in proportion 1:1.

### 2.4 Tumor Tissue Processing

Fresh tumor samples were enzymatically digested in a mixture containing RPMIc (Roswell Park Memorial Institute Medium (Thermo Fisher Scientific; Cat. # 11875085 ) supplemented with 10% fetal bovine serum (PAN‐Biotech; Cat. # P30‐3031) and 100 U/mL penicillin–streptomycin (Thermo Fisher Scientific; Cat. # 15140122 )) further supplemented with 0.2 µg/mL dispase (Sigma–Aldrich; Cat. # D4693‐1G), 0.2 µg/mL collagenase A (Roche; Cat. # 11088866001), and 100 µg/mL DNase I (Sigma–Aldrich; Cat. # DN25‐100MG). Digestions were performed using 1 mL of enzyme solution per sample for 45 min, shaking at 500 rpm and 37°C. The cell suspensions were filtered through 100 µm sterile strainers (Sysmex Europe GmbH; Cat. # 04‐004‐2328) and centrifuged at 300 × g and 4 °C for 5 min, after which the supernatant was removed. Pellets were dissolved in RPMIc.

### 2.5 Flow Cytometry

#### 2.5.1 Healthy Donor and Stem Cell Transplantation Blood

For determination of CD177 expression pattern on human neutrophils 5 × 10^5^ isolated neutrophils were stained with APC conjugated anti‐CD177‐antibody (Miltenyi Biotec, Cat. # 130‐123‐836) and VioBright V600 conjugated anti‐CD66b‐antibody (Miltenyi Biotec, Cat. # 130‐132‐148). In brief, cells were resuspended in 100 µL MACS‐running‐buffer (Miltenyi Biotec, Cat. # 130‐092‐747) containing 2 µL of each respective antibody (following concentrations given by the supplier). Samples were incubated for 15 min at 4°C, washed by adding 150 µL buffer, centrifuged at 500xg for 5 min and resuspended in 200 µL buffer. Events were gated for cells, singlets, CD66b positive events and lastly CD177 positive cells. In total 2 × 10^5^ events per sample were recorded. All steps were carried out at 4°C.

For intracellular staining of CD177 1 × 10^6^ isolated neutrophils were stained as described above for surface staining. Afterward cells were washed once with buffer. Cells were fixed and permeabilized using Foxp3 / Transcription Factor Staining Buffer Set (eBioscience, Cat. # 00‐5523). In brief cells were fixed in Fixation/Permeabilization Buffer for 30 min at 4°C, followed by two washing‐steps in 1x Permeabilization Buffer (PB). FITC conjugated anti‐CD177 antibody (Miltenyi Biotec, Cat. # 130 126 380) was prepared in PB and cells stained for 30 min at 4°C, protected from light. Samples were washed 2x with PB before resuspending in FACS‐buffer and events were acquired on a MACSQuant Analyzer 16 Flow Cytometer.

#### 2.5.2 Stroke Patient Blood

Venous blood was collected in EDTA tubes at three different timepoints post admission to the Stroke Unit of the University Hospital Essen (T1: 0–72 h; T2: 72–144 h; T3: 90 d). Isolated blood was processed with BD Pharm Lyse buffer (10 mL per 1 mL blood), for 15 min protected from light and centrifuged at 900xg for 5 min at 4°C. Pellets were resuspended in PBS with 1% FCS. Following antibodies were used: anti‐CD66b FITC (Clone 80H3, Beckman Coulter, Cat. # IM0531U), 7‐AAD PE‐Cy5.5 (Beckman Coulter, Cat. # A07704), anti‐CD45 BV785 (Clone HI30, BioLegend, Cat. # 304048), anti‐CD15 BV605 (Clone W6D3, BioLegend, Cat. # 323032), anti‐CD62L PE (Clone DREG56, Beckman Coulter, Cat. # IM2214U), and anti‐CD177 APC (Clone REA258, Miltenyi Biotec, Cat. # 130‐123‐836). Cells were incubated for 20 min at 4°C in the dark. Afterward samples were adjusted to 100 µL using PBS with 1% FCS. Cells were characterized on a CytoFLEX S (Beckman Coulter) and subsequently analyzed by Kaluza Analysis 2.1 software (Beckman Coulter) employing the following gating strategy: Cells→Singlets→CD45^+^/7‐AAD^−^ (viable leukocytes)→CD66b^+^/CD15^+^ (Neutrophils)→CD66b^+^/CD177^+^.

#### 2.5.3 HNC Patient Blood and Tumor Tissue

Cells isolated from HNC patient material or healthy donor controls, as described above were washed with PBS, resuspended in PBS (Thermo Fisher Scientific; Cat. # 14190136) containing human FC block (BD Bioscience; Cat. # 564220), stained with anti‐huCD45 VioGreen (Miltenyi Biotec; Cat. # 130‐113‐124), anti‐huCD66b FITC (Beckman Coulter; Cat. # IM0531U), anti‐huCD177 APC (Miltenyi Biotec, Cat. # 130‐123‐836), viability dye eFluor 780 (eBioscience; Cat. # 564220), all used at dilutions recommended by the respective vendor. Cells were incubated at 4 °C for 30 min, afterward cells were washed 1x with PBS (Gibco; Cat. # 10010023) and events recorded on a BD FACSCanto II Clinical Flow Cytometry System.

### 2.6 Neutrophil Sorting

#### 2.6.1 FACS

For experiments involving fluorescent‐activated cell sorting (FACS) total cell numbers of isolated neutrophils were determined and stained with APC conjugated anti‐CD177‐antibody (Miltenyi Biotec, Cat. # 130‐123‐836) following the suppliers recommended dilution. Cells were incubated for 15 min at 4°C, washed with DPBS (PAN‐Biotech; Cat. # P04‐36500) and resuspended in adequate amount of DPBS, to ensure acceptable cell‐density. Cell separation was carried out using a BD Aria III Cell Sorter, applying pre‐gating on single cells and strict gating for CD177 negative and positive subpopulations.

#### 2.6.2 MACS

For experiments involving magnetic cell separation (MACS) freshly isolated neutrophils were first stained with biotin coupled anti‐CD177‐antibody (Miltenyi Biotec, Cat. # 130‐101‐529) using recommended amounts based on total cell numbers as per the supplier's instructions, for 20 min at 4°C. Following a washing step, marked cells were magnetized using Streptavidin Microbeads (Miltenyi Biotec, Cat. # 130‐048‐101) following manufacturer's protocol. Mixed input has been separated into labeled cells and unlabeled cells by passing them through a LS column (Miltenyi Biotec, Cat. # 130‐042‐401), collecting both the flow‐through and flushed out content (designated CD177 enriched population). To ensure purity ≥98 % the flow‐through was passed over an additional LD column (Miltenyi Biotec, Cat. # 130‐042‐901), collected secondary flow‐through was designated as CD177 depleted subpopulation.

### 2.7 In Vitro Stimulation

5 × 10^5^ freshly isolated neutrophils were resuspended in 200 µL X‐vivo 10 (Lonza, Cat. # BP04‐743Q; LOT:0001151113) supplemented with 1x Serum Replacement 3 (Sigma Aldrich, Cat. # S2640‐10ML). All respective stimulants listed in Table 1 were used in a final concentration of 50 ng/mL. Cells were incubated for 1 h at 37°C with 5% CO_2_. Following incubation, cells were stained as described above for flow cytometry with the inclusion of an anti‐CD62L antibody conjugated to VioGreen (Miltenyi Biotec, Cat. # 130‐130‐549).(Table 1)

### 2.8 Real‐Time Quantitative Polymerase Chain Reaction

RNA of FACS sorted, circulating human neutrophils from 9 healthy donors was extracted via an RNA isolation kit (Qiagen, Cat. # 74104) according to the manufacturer's instructions. Obtained RNA was eluted in 30 µL, a total of 100 ng was transcribed to cDNA using a High‐Capacity cDNA Reverse Transcription Kit (Applied Biosystems, Cat. # 4368814) in a peqSTAR 96X Universal Gradient thermocycler. Total volume was made up to 120 µL with UltraPure DNase/RNase‐Free Distilled Water per sample. To 4 µL of transcribed cDNA, 10 µL TaqMan Fast Advanced Master Mix (Applied Biosystems, Cat. # 4444557), 5 µL UltraPure DNase/RNase‐Free Distilled Water and 1 µL of the respective TaqMan probe were added. We used the following probes from Thermo Fisher Scientific: GAPDH (Hs02786624_g1), CD177 (Hs00360669_m1), CXCR2 (Hs01891184_s1), CXCR4 (Hs00607978_s1), CXCL8 (Hs00174103_m1). Gene expression was measured on a CFX96 Touch Real‐Time PCR Detection System (Bio‐Rad, Cat. # 185–5195) using the following conditions: 1 × 50°C for 2 min, 1 × 95°C for 2 min, 40 cycles of 95°C for 1 s, and 60°C for 20 s. Reactions were performed in triplicates and analyzed via Δ‐Δ‐ct method.

### 2.9 Exploratory Analysis of Single‐cell RNA Sequencing Data

Raw sequencing data from publicly available human bone marrow samples were obtained from ArrayExpress (E‐MTAB‐11188) [13]. FASTQ files were processed using CellRanger v7.0 pipeline with default settings to align reads and generate feature‐barcode matrices. Quality control and filtering were performed in R using DropletUtils and scater packages, following standard workflows to remove low‐quality cells, potential doublets and cell‐free RNAs. Normalization and variance modeling were carried out in scater. PCA and UMAP were calculated with corresponding functions and default parameters from scater package with additional batch removal using batchelor and harmony. Marker gene identification was performed with scater's scoreMarkers function, which calculates effect sizes and ranks features across groups to identify cluster‐specific gene signatures.

### 2.10 Confocal Microscopy

Glass coverslips placed in 24‐well plates were pre‐coated with poly‐L‐lysine (Sigma–Aldrich; Cat. # P4832‐50ML) for 30 min at 37°C. Isolated neutrophils were resuspended in X‐vivo 10 (Lonza, Cat. # BP04‐743Q; LOT:0001151113) supplemented with 1x Serum Replacement 3 (Sigma Aldrich, Cat. # S2640‐10ML) and seeded onto coverslips. Cells were cultured at 37°C for 1 h to allow adherence, washed once with DPBS (PAN‐Biotech; Cat. # P04‐36500) and fixed in 2% paraformaldehyde for 10 min at RT. After washing twice with DPBS, cells were blocked in 2% BSA (Sigma–Aldrich; Cat. # A9418‐50G) in DPBS for 2 h at RT. Following, cells were stained with APC conjugated anti‐CD177 antibody, using 2 µL per sample (Miltenyi Biotec, Cat. # 130‐123‐836) and FITC conjugated anti‐CD66b antibody, using 5 µL per sample (Invitrogen, Cat. # 11‐0666‐42) diluted in 2% BSA/DPBS to a total volume of 100 µL per well, for 2 h at RT, in the dark. Cells were washed three times with DPBS for 5 min each and counterstained with DAPI (diluted 1:5000 in DPBS) (Thermo Fisher Scientific, Cat. # 62248) for 10 min at RT. Afterward, coverslips were washed again once with DPBS and mounted on glass slides using Fluoromount (SouthernBiotech, Cat. # 0100‐01).

Fluorescence images were acquired using a Leica SP8 confocal microscope equipped with a HC PL APO CS2 40X/1.3 oil immersion objective. To excite DAPI, FITC and APC, a 405 nm diode laser and a 488 and 633 nm Withe Light Laser (WLL) were used. Bandpass filters 418‐492, 501‐551 and 640–720 nm were used for emission detection. Images were acquired in sequential mode and consisted of 15 z‐sections, with voxel sizes at 0.104×0.104×0.346 µm (x,y,z) and 2.74‐times zoom. Antibody isotype controls and unstained cell samples were included to check for unspecific antibody binding and background fluorescence.

### 2.11 Morphological Feature Analysis

Sorted neutrophils were prepared via cytospin. Per slide 6000 neutrophils were spun onto slides for 5 min at 1450 rpm using the Cytospin 4 Cytocentrifuge (Thermo Fisher Scientific) in a 1:1 mixture of DPBS (PAN‐Biotech; Cat. # P04‐36500) to FBS (Capricorn Scientific; Cat. # FBS‐12A)(total volume per slide 400 µL). Afterward, cells were fixed for 10 min at RT in a 7:1 mixture of Ethanol (Carl‐Roth; Cat. # 9065.1) to 37% Formaldehyde solution (Carl‐Roth; Cat. # 4979.1), washed 2x in H_2_O, before being subjected to May‐Grünwald‐staining. Slides were imaged using the EVOS M7000 Imaging system (Thermo Fisher Scientific; Cat. #AMF7000).

For subsequent analysis of morphological hallmarks of neutrophils sorted by CD177 expression status, we developed a full microscopy image analysis pipeline, as depicted in Figure S2. A Cellpose model was used to delineate the boundaries of cells (instance segmentation), in addition ilastik pixel classifier had been trained to segment all pixels of nuclei (semantic segmentation). Segmentation was used to generate single cell images with superimposed cell‐ and nuclear‐masks for feature extraction and downstream statistical analysis. A total of 80 features were computed, resulting 20 different metrics (see Table 2). Features of all 4185 analyzed cells were visualized via dimension reduction with both UMAP and t‐SNE. All measured features were additionally examined for statistically significant differences between annotated groups, examining p‐value and effect size (absolute r‐value).

**TABLE 1:** Stimulants used for neutrophil activation in vitro.

Stimulant	Supplier	Cat. #
ATP	Roche	10127523001
LTB4	Tocris	2307
fMLP	Sigma–Aldrich	3506‐5MG
LPS	Sigma–Aldrich	L8274‐10MG
TNF‐α	Cell Signaling Technology	16769
IL‐8 (R&D 208‐IL‐010/CF)	R&D Systems	208‐IL‐010/CF
IL‐1β	MedChemExpress	HY‐P7028
IL6	MedChemExpress	HY‐P7044
GM‐CSF	MedChemExpress	HY‐P78868
CytochromC	MedChemExpress	HY‐125857
C5α	Sino Biological	10604‐HNAE
IL‐17	Sino Biological	12047‐HNAE
G‐CSF	Ratiopharm	151443

**TABLE 2:** List of 20 metrics used in feature calculation. The 20 metrics were calculated on each cell, which result in 80 features in total.

Index	Feature name	Description
Morphology measurement		
1	size	Number of pixels in the region
2	convex_size	Number of pixels in the convex hull of the region
3	eccentricity	Ellipse eccentricity fitted to region; value between 0 and 1, with 0 being a circle, near‐1 being extremely elongated
4	Equivalent_diameter	Diameter of a circle with the same area as the region
5	Euler_number	Components minus holes in the region (topology measure)
6	extent	size divided by bounding box size (i.e., compactness within the bounding box)
7	filled_area	Area after filling holes in the region
8	major_axis_length	Length of the major axis of the best‐fit ellipse
9	minor_axis_length	Length of the minor axis of the best‐fit ellipse
10	axis_ratio	minor_axis_length / (major_axis_length + 1e‐8); measurement of roundness / elongation.
11	perimeter	Length of the region boundary (in pixels)
12	solidity	size / convex_size; how convex (1 means fully convex)
Texture measurement		
13	contrast	Weighted intensity difference between neighboring pixels, ∑(i−j)^2^ P(i,j). Higher = sharper/local edges; lower = smoother texture
14	dissimilarity	Absolute intensity difference, ∑|i−j| P(i,j). Higher = more gray‐level variation; lower = more uniform
15	homogeneity	Closeness of distribution to the diagonal, ∑ P(i,j)/(1+(i−j)^2^). Higher = more uniform/coarse; lower = more contrasty.
16	energy	Magnitude of repeated patterns, √(∑ P(i,j)^2^). Higher = more regular/repetitive texture; lower = more random
17	ASM	Angular Second Moment, ∑ P(i,j)^2^. Squared version of energy (i.e., energy^2^), same interpretation
18	glcm_dispersion	Standard deviation of the GLCM entries measuring spread/heterogeneity of co‐occurrence probabilities. Higher = more diverse co‐occurrences; lower = more concentrated/regular
Crowdness measurement		
19	mean_crowdedness	for each lobes’ centroid, compute Euclidean distances to all centroids; take the k nearest (default k = 10), including self; then take the mean of these k+1 distances (this feature is only applicable for nuclei, set to 0 when applied on others)
20	std_crowdedness	The standard deviation of above k+1 distance (this feature is only applicable for nuclei, set to 0 when applied on others)

### 2.12 Proteomics Comparison

Neutrophil pellets (20,000 cells/pellet) were lysed in 1% SDS buffer, sonicated for 10 min using a Bioruptor (Diagenode), and treated with Benzonase (Merck Bioscience; Cat. # 70664) at 37°C for 30 min. Protein concentration was determined using the BCA assay (Thermo Scientific; Cat. # 23225). Subsequently, proteins were reduced with 10 mM dithiothreitol (DTT) (Sigma Aldrich; Cat. # D9779) for 30 min at 56°C and alkylated in the dark with 20 mM iodoacetamide (IAA) (Sigma–Aldrich; Cat. # I1149) for 30 min. Proteolytic digestion was carried out using S‐Trap micro spin columns (Protifi), as described previously [14]. After digestion, tryptic peptides eluted from the columns were vacuum dried and reconstituted in 0.1% trifluoroacetic acid (TFA) (Biosolve; Cat. # 00202341A8BS). Equal amounts of peptides from each donor and each condition were analyzed on a timsTOF‐HT mass spectrometer (Bruker Daltonics) coupled to an UltiMate 3000 nano‐LC system (Dionex). Peptides were first trapped on PepMap Neo Trap Cartridge (Thermo Scientific, Catalog number 174500) and subsequently separated on C‐18 reversed‐phase Aurora column (25 cm length X 75 µm inner diameter) equipped with an integrated emitter (Ion Opticks). Chromatographic separation was achieved using a 90‐min linear gradient ranging from 3% to 35% buffer B (84% acetonitrile, 0.1% formic acid in water). Data was acquired in DIA PASEF mode using long gradient high sensitivity method covering precursor isolation window of 400–1200 m/z and ion mobility (1/K0) range of 1.6 to 0.6 Vs cm−2. The mass width was at 26 Da with an overlap of 1 Da, resulting in total 32 mass scan events and 16 MS/MS ramps. The collision energy was adjusted at 59 eV at 1/K0  =  1.6 Vs cm−2 and decreasing to 20 eV at 1/K0  =  0.6 Vs cm−2. Ion accumulation and TIMS ramp time were set at 100 ms, yielding a cycle time of 1.8 s. High sensitivity detection was enabled.

The raw data were analyzed on Spectronaut v.19 (Biognosys) using directDIA approach with the Pulsar search engine against UniProt database (UP000005640, 20 404 sequences, downloaded on January 30, 2023). Default Biognosys settings were applied, including trypsin/P specificity, a maximum of two missed cleavages, carbamidomethylation of cysteine as a fixed modification, and methionine oxidation as a variable modification. For label‐free quantification, only precursors passing a Q‐value threshold of ≤0.01 were considered. The MaxLFQ algorithm was used to quantify proteins, allowing at least three peptides per protein. Data normalization was achieved through a local normalization strategy based on retention time (RT)‐dependent logistic regression models across the run. Statistical analysis was carried out in MetaboAnalyst v5. Log2‐transformed intensity values at MS2 levels were used for paired t‐tests. Proteins showing a log2 fold change (positive or negative) ≥1 and an FDR‐adjusted p‐value <0.05 were considered statistically significant. Pathway analysis was performed using DAVID (DAVID Knowledgebase v2024q4).

### 2.13 Lipidomic Profiling

#### 2.13.1 Materials and Nomenclature

Acetonitrile (ACN), methanol (MeOH), and water (HiPerSolv CHROMANORM) were obtained from VWR International GmbH (Darmstadt, Germany). Isopropanol (IPA) was purchased from Carl Roth GmbH + Co. KG (Karlsruhe, Germany). Methyl tert‐butyl ether (MTBE) was acquired from Merck (Darmstadt, Germany). Ammonium formate (NH4HCO2) and formic acid (FA) were obtained from Sigma–Aldrich. EquiSPLASH was purchased from Avanti Polar Lipids (Alabaster, AL, USA). Lipid nomenclature is following the LIPID MAPS shorthand nomenclature [15].

#### 2.13.2 Lipid Extraction

All neutrophil pellets were re‐suspended in PBS containing butylated hydroxytoluene (BHT) and kept on ice. A volume of 173.9 µl ice‐cold MeOH containing 5 µL EquiSplash were added into each sample. Subsequently, 625 µL of MTBE were added, and the samples were incubated for 1 h at 4°C while shaking at 750 rpm. To induce phase separation, 156 µL of water were added, and the samples were incubated for 20 min at 4°C while shaking at 750 rpm. The homogenates were centrifuged at 4°C and 16,000×g for 4 min. The upper organic phase was transferred to an Eppendorf tube and dried using a SpeedVac vacuum concentrator (SPD120, Thermo Fisher Scientific, Bremen, Germany). The dried extracts were stored at −80°C until further use.

#### 2.13.3 Lipidomics LC‐MS Measurements

Neutrophils were reconstituted in 100 µL of IPA and transferred into LC‐MS vials. Reversed‐phase liquid chromatography mass spectrometry (RPLC‐MS) was performed on a Vanquish Flex UHPLC‐system (Thermo Fisher Scientific, Dreieich, Germany) equipped with a C18 guard and analytic column (YMC Triat C18, 150 × 2.1 mm, 1.9 µm, 120 Å, Dinslaken, Germany) coupled to an Exploris 240 orbital trapping mass spectrometer (Thermo Fisher Scientific, Bremen, Germany) equipped with a HESI probe. In positive‐ion mode 5 µL and in negative‐ion mode 10 µL were injected into the UHPLC. The gradient for separation was: 0–3 min from 10% to 25% B, 3–9 min from 25% to 54% B, 9–14 min from 54% to 77% B, 14–18 min from 77% to 88% B, 18–22 min from 88% to 95% B, 22–30 min at 95% B, 30–30.5 min from 95% to 100% B, and 30.5–38 min at 100% B. Mobile phase A consisted of ACN/water, 1:1, v/v and mobile phase B was composed of IPA /ACN/water, 85:10:5, v/v/v. Both phases contained 5 mM NH4HCO2 and 0.1% FA. A flow rate of 300 µL/min as well as a column oven temperature of 50°C were employed for all separations. The mass spectra were recorded in both positive‐ and negative‐ion modes using the following ESI conditions: sheath gas, 40 L/min; auxiliary gas, 10 L/min; sweep gas, 1 L/min; spray voltage, +3.5 kV (positive‐ion mode) and –2.5 kV (negative‐ion mode); capillary temperature, 300°C; S‐lens RF level, 35; and auxiliary gas heater temperature, 370°C.

Data were acquired in positive‐ and negative‐ion modes (m/z 200–1200) at a resolution of 120 000 (referenced at m/z 200). The AGC target of 1 × 10^6^ and a 100 ms maximum injection time was applied for all measurements. All runs utilized internal mass calibration via Easy‐IC

Tandem mass spectra were acquired in data‐dependent acquisition (DDA) mode with a cycle time of 1.3 s at a resolution of 15 000 (at m/z 200) in both ion modes. The DDA parameters were as follows: AGC target, 1 × 10^5^; maximum injection time, 60 ms; isolation window, 1.2 m/z; stepped NCE, 17/27/37; and charge state, 1. Isotope exclusion was enabled, and dynamic exclusion was set to 6 s (±2.5 ppm tolerance, exclusion after 2 detections within 6 s).

Lipids were identified using Lipostar2 (v2.1.7, MassAnalytica, Sant Cugat del Vallés, Spain) with an MS1 and MS2 mass accuracy tolerance of ±5 ppm and ±10 ppm, respectively. All other parameters were kept as default. Annotations were manually verified and filtered by plotting retention time against hydrogen‐based Kendrick mass defects to remove outliers. Lipid species that deviated from expected trendlines were excluded. The area‐under‐the‐curve (AUC) of identified lipids was normalized to the corresponding spiked lipid standards and expressed as mol%. Statistical analysis and volcano plots were performed in MetaboAnalyst (v.6.0) and replotted in GraphPad Prism (v8.4.3, Boston, MA, USA).

### 2.14 Viability Assay

Viability of MACS sorted blood derived neutrophils was investigated using a commercial fluorescent apoptosis/necrosis detection kit (Abcam, ab176749), staining for 7‐AAD (necrosis), Apopxin (apoptosis) and CytoCalcein (viable). According to manufacturer's instruction kit components were added to neutrophils in pre‐warmed X‐Vivo 10 (Lonza, Cat. # BP04‐743Q), supplemented with 0.3x serum replacement 3 (Sigma Aldrich, Cat. # S2640‐10ML) into a 96‐well plate. Time‐laps recordings of cells were taken with 5 min intervals on an inverse DMI8 Leica Thunder system, fitted with an 20x air objective. Fluorophores were excited at 390 nm for CytoCalcein Violet, 475 nm for Apopxin Green, and 555 nm for 7‐AAD, and subsequently filtered through a bandpass filter at 420–500, 500–570, and 602–682 nm, respectively.

An automated apoptosis detection and counting algorithm (ADAPT, Automated Detection of Apoptosis by Processing and Tracking) was used to differentiate cell state over time in single channel fluorescent and brightfield images. Images of selected timepoints were thresholded for background subtraction. Automated particle counting based on particle size was used for determining total cell numbers per field of view (FOV), additional, noncircular structures were excluded from the analysis. Viable cells were defined as the proportion of CytoCalcein positive cells (fluorescent channel) out of total cells (brightfield channel) per FOV. An executable version of the used script with detailed documentation can be accessed at: https://github.com/Deuto94/ADAPT

### 2.15 ROS Production Assay

For analysis of reactive oxygen species (ROS) production, 5 × 10^5^ human neutrophils per sample were incubated with 5 µM dihydrorhodamine‐123 (DHR‐123) (Genaxxon Bioscience; Cat. # S5427.0002) in Hank's balanced salt solution (Bio West; Cat. # L0605) containing 20 mM HEPES (AppliChem; Cat. # A1069), 0.25% bovine serum albumin (Sigma–Aldrich; Cat. # A6003), 0.1% glucose (Merck; Cat. # 108342), and 1.2 mM Ca^2+^ (AppliChem; Cat. # A4088) for 10 min at 37°C. Then neutrophils were stimulated with 50 ng/mL phorbol 12‐myristate 13‐acetate (PMA) (Merck; Cat. # 524400) or left unstimulated in presence of 0.05% dimethyl sulfoxide (DMSO) (Th. Geyer; Cat. # 2347.1000) for 20 min at 37°C. Afterward, the cells were immediately placed on ice, centrifuged for 5 min at 300xg at 4°C, resuspended in LIVE/DEAD Fixable Violet Dead Cell Stain (Invitrogen; Cat. # L34955) in PBS, and incubated on ice for 15 min before addition of an APC‐conjugated anti‐human CD177 antibody (Miltenyi Biotec Cat. # 130‐101‐511) for 15 min at 4°C in PBS containing 0.5% BSA and 2 mM EDTA (AppliChem; Cat. # A4892). Cells were fixed with 2% formaldehyde in PBS for 15 min on ice and resuspended in PBS containing 0.5% BSA and 2 mM EDTA for analysis using a CytoFLEX S flow cytometer (Beckman Coulter). 20 000 events were recorded per sample and data analysis was performed with FlowJo 10 software (BD Biosciences). The median fluorescence intensity of DHR‐123 as a measure of ROS production was calculated from CD177‐negative and ‐positive neutrophils, gated according to their APC signal.

### 2.16 Phagocytosis Assay

To analyze bacterial uptake, *Escherichia coli* MG1655 carrying the plasmid pEB2‐E2‐Crimson were cultured in LB medium (Carl Roth; Cat. # X964.1) containing 50 µg/mL kanamycin (Sigma–Aldrich; Cat. # 60615) over night at 37°C shaking at 180 rpm. Bacteria were diluted to an OD_600_ of 0.01 and cultured further for approximately 2 h at 37°C shaking at 180 rpm. 2 × 10^5^ human neutrophils per sample were incubated with *E. coli* MG1655 pEB2‐E2‐Crimson at a multiplicity of infection (MOI) of 10 for 15 min at 37°C and 5% CO_2_ in RPMI 1640 (Sigma–Aldrich; Cat. # R8758) containing 10% fetal calf serum (FCS) (Sigma–Aldrich; Cat. # F7524). Cells were placed on ice, centrifuged for 5 min at 300xg at 4°C, resuspended in LIVE/DEAD Fixable Violet Dead Cell Stain (Invitrogen; Cat. # L34955) in PBS, and incubated on ice for 15 min. A FITC‐conjugated anti‐human CD177 antibody (Miltenyi Biotec; Cat. # 130‐126‐380) was added for 15 min at 4°C in PBS containing 0.5% BSA and 2 mM EDTA (AppliChem; Cat. # A4892) before fixation with 2% formaldehyde in PBS for 15 min on ice. Cells were resuspended in PBS containing 0.5% BSA and 2 mM EDTA and analyzed using a CytoFLEX S flow cytometer (Beckman Coulter). 30 000–50 000 events were recorded per sample and data analysis was performed with FlowJo 10 software (BD Biosciences). The median fluorescence intensity of E2‐Crimson as a measure of *E. coli* taken up by the cells was calculated from CD177‐negative and ‐positive neutrophils gated according to their FITC signal. pEB2‐E2‐Crimson was a gift from Philippe Cluzel (Addgene plasmid # 104010; http://n2t.net/addgene:104010; RRID:Addgene_104010) [16].

### 2.17 Sterile Irritation Mimicking Transmigration Assay

Transmigration of neutrophils was induced in five healthy human individuals as previously described [14] by washing their mouths with a Tabasco solution (10% in saline) for 30 s. After 2 h the oral cavity was washed three times with saline solution, collecting sputum and filtered through 30 µm pore size mesh filters. Blood from the same donors was collected in EDTA tubes, and erythrocytes were lysed for 20 min on ice using RBC lysis buffer (BioLegend, Cat. # 420302). For subsequent flow cytometric analysis, unwanted FcR‐involved staining was blocked using Human TruStain FcX (BioLegend, Cat. # 422302) before cells were stained with anti‐CD45 (BioLegend; Cat. # 103127), anti‐CD66b (BioLegend; Cat. # 305103 or 305112), anti‐CD15 (BioLegend; Cat. # 702057), anti‐CD11b (BioLegend; Cat. # 301327), anti‐CD62L (BioLegend; Cat. # 304805), and anti‐CD177 (BioLegend, Cat. # 315804 or 315808). Data has been acquired on a Cytek Aurora (5L, Cytek Biosciences), and analyzed using FlowJo 10 (BD Bioscience).

### 2.18 Tumor Cell Killing Assay

Isolated neutrophils and neutrophils‐T cells mixtures were co‐incubated with UT‐SCC‐50 tumor cells in the ratio 10:10:1 (100 000 neutrophils, 100 000 T cells to 10 000 tumor cells) in 200 µL of RPMIc in a 48‐well plate in duplicate for each biological replicate and incubated for 48 h at 37°C, 5% CO_2_. After incubation, the wells were washed gently with PBS to remove neutrophils, T cells, and dead tumor cells.

To evaluate the morphological changes of the tumor cells driven by neutrophils, well‐alive adherent tumor cells remaining in the culture were fixed with 1 mL of ice‐cold 96% methanol for 1 h at −20°C, followed by staining with 0.01% aqueous solution of gentianine (Santa Cruz, Cat. # CAS 439‐89‐4) for 10 min. The plate was washed with dH_2_O and left to dry overnight. The amount and morphology of tumor cells after co‐incubation with neutrophils and T cells were evaluated using AMG EVOS digital phase‐contrast inverted microscope and analyzed using FIJI (ImageJ) software (https://fiji.sc/).

### 2.19 Analysis of Tumor Associated Neutrophils

Formalin‐fixed, paraffin‐embedded (FFPE) tumor tissue microarrays (TMAs) from 26 patients (Table 3) were obtained and processed for immunofluorescence analysis. Sections were deparaffinized through a graded alcohol series (two times Xylene for 5 min, two times 2‐propanol for 3 min, 100%, 96% and 70% ethanol once for 3 min) and rehydrated in distilled water. Autofluorescence was quenched by incubation with Sudan Black (2% in 70% Ethanol) for 15 min at room temperature (RT), followed by rinsing in phosphate‐buffered saline (PBS). To reduce non‐specific binding, the sections were blocked with bovine serum albumin (5% in PBS) for 20 min at RT.

**TABLE 3:** Clinicopathological characteristics of human tumor tissue analyzed for tumor‐associated neutrophils (TANs).

	Tumor tissue (n = 26)
Age (years)	61 (42‐92)
Sex, male (N, %)	21 (80.8%)
Localization:	
Nasopharynx	6 (23.1%)
Hypopharynx	20 (76.9%)
T Stage:	
1 (N,%)	1 (3.8%)
2 (N,%)	8 (30.8%)
3 (N,%)	6 (23.1%)
4 (N,%)	11 (42.3%)
N Stage:	
0 (N,%)	9 (34.6%)
1 (N,%)	3 (11.5%)
2(N,%)	14 (53.9%)
UICC:	
I (N,%)	0
II (N,%)	4 (15.4%)
III (N,%)	4 (15.4%)
IV (N,%)	18 (69.2%)
Co‐morbidities:	
Arterial hypertension	17 (65.4%)
Coronary artery disease	6 (23.1%)
Heart failure	4 (15.4%)
Diabetes mellitus	6 (23.1%)
COPD	5 (19.2%)
Obesity (BMI >30 kg/m^2^)	5 (19.2%)
Nicotine abuse	25 (96.2%)
Alcohol abuse (>2 drinks per day)	4 (15.4%)
Medications	
ACE inhibitors	14 (53.8%)
Beta‐blockers	8 (30.8%)
Anticoagulants	6 (23.1%)
Metformin	5 (19.2%)
Statins	4 (15.4%)

Immunofluorescence staining was performed using primary antibodies against CD66b (diluted 1:100 in PBS) (BioLegend; Cat. # 396902) and CD177 (1:200 in PBS) (OriGene; Cat. # TA349765). Incubation with primary antibodies was carried out at RT for 2 h, followed by washing in PBS and TBS. Secondary antibody staining was performed with Alexa Fluor 546‐conjugated anti‐mouse IgG (1:200 in PBS) (Invitrogen; Cat. # A10036) and Alexa Fluor 750‐conjugated anti‐rabbit IgG (1:200 in PBS) (Invitrogen; Cat. # A21039). Incubation with secondary antibodies was carried out at RT for 1 h, followed by washing with PBS. DAPI (1:200 in PBS) (BioLegend; Cat. # 422801) staining was performed with an incubation time of 30 min.

Fluorescence images were acquired with an Axioscan Z1 slide scanner (Carl Zeiss) at the Imaging Center (IMCES), University Hospital Essen. Raw image data were subjected to background subtraction using Imaris software (Version 10.1). Quantitative analysis of stained cell populations and tissue compartments was performed with QuPath (Version 0.5.1) and the StarDist extension.

### 2.20 Bone Marrow Transfer Experiments

Peripheral blood was taken at donor‐screening, before conditioning chemotherapy and at day +28 after stem cell transplantation. Patients and Donors gave informed consent. Human biological samples and related data were provided by the Westdeutsche Biobank Essen (WBE, University Hospital Essen, University of Duisburg‐Essen, Essen, Germany; approval 21‐10271‐BO). Successful engraftment was defined as absolute neutrophil count ≥500 and platelet levels ≥20.000 per µl blood. Remission was verified via diagnostic bone marrow analysis on day +28. Chimerism was determined via Next‐Generation‐Sequencing (NGS) at a sensitivity level of <0.1% for recipient features.

### 2.21 Statistical Analysis

Unless otherwise stated, continuous data are expressed as mean±SD for normally distributed data. For statistical analysis of normally distributed, single factor data one‐way ANOVA was used for comparison, for multiple comparison two‐way ANOVA was chosen. Simple linear regression models were tested for significant differences using two‐way ANOVA. Non‐normally distributed data were analyzed via Mann–Whitney‐U test. Survival curves were analyzed with both Log‐rank (Mantel–Cox) and Gehan–Breslow–Wilcoxon test. P‐values ≤0.05 were considered statistically significant. Statistical analysis was performed using GraphPadPrism (La Jolla, USA).

## 3 Results

### 3.1 CD177^−^ Neutrophil Blood Counts are Stable Under Homeostasis and Activation

**FIGURE 1:**
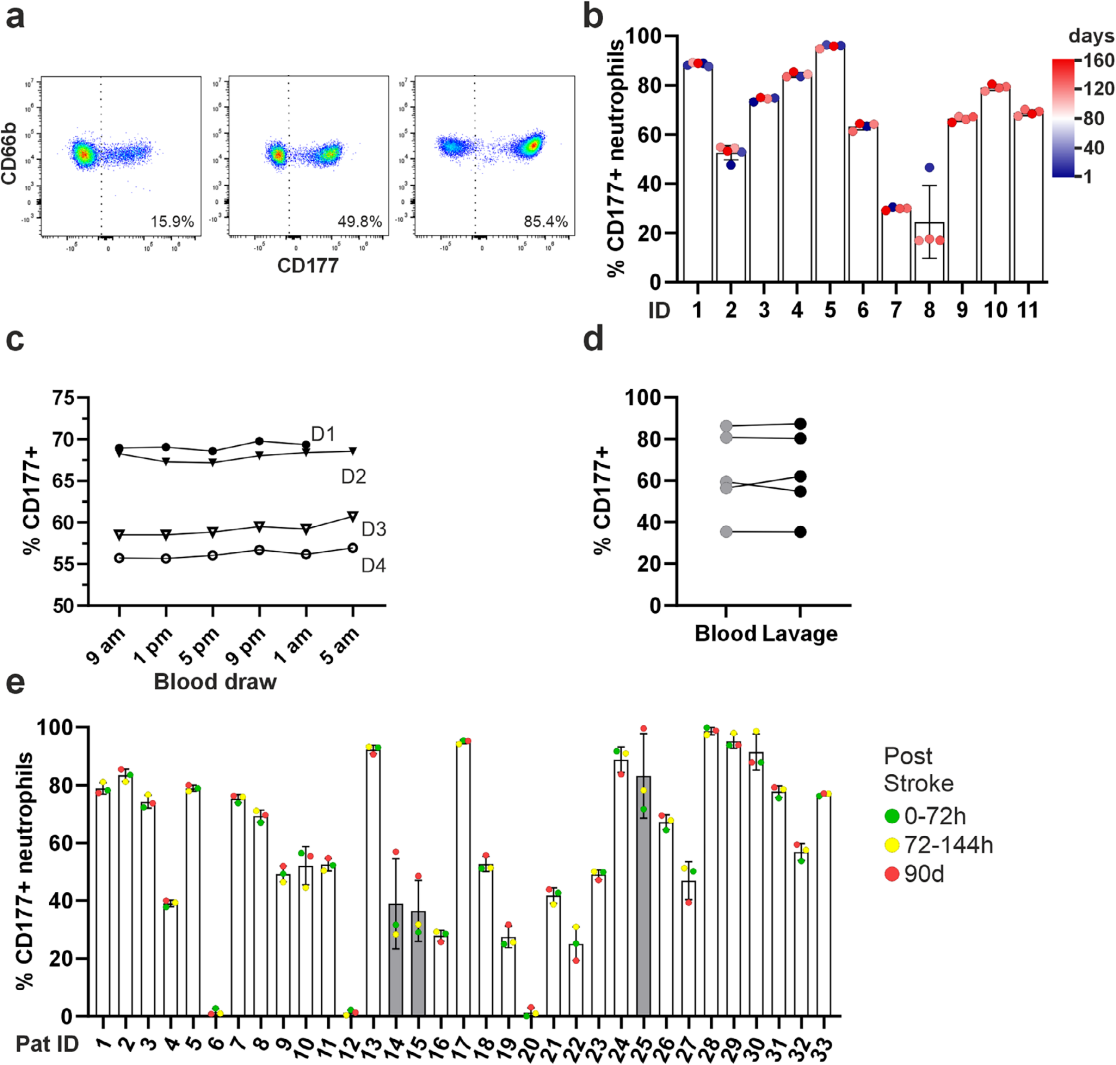
CD177 marks a stable and durable neutrophil subpopulation. (a) Exemplary flow cytometry plots showing differential CD177 expression on neutrophils (CD66b^+^) in three individuals. (b) Percentage of CD177‐expressing neutrophils out of total neutrophil count in 11 healthy donors measured consecutively over multiple days, up to 160 days apart, values represented as mean±SD, with individual values color‐coded for the day of measurement. (c) Percentage of CD177^+^ neutrophils out of total neutrophil count in 4 healthy donors, measured over the course of 24 h at different timepoints. (d) Proportion of CD177^+^ neutrophils found in blood or sputum of 5 healthy individuals 2 h after irritation of the oral cavity using a 10% Tabasco‐saline solution. (e) Proportion of circulating CD177^+^ neutrophils in 33 ischemic stroke patients at different timepoints post hospital admission, measured via flow cytometry of peripheral blood. Grey bars depict patients with changes ≥18% over time; data presented as mean±SD for individual values at indicated time points.

To elucidate the dynamics of CD177 expression in circulating neutrophils, we investigated blood from healthy individuals using flow cytometry and confirmed different patterns of CD177^+^ and CD177^−^ (Figure 1a). Measuring multiple individuals repeatedly showed that the CD177^+^/CD177^−^ ratio of mature blood neutrophils was stable over at least 160 days (Figure 1b). Also, when observing individuals repeatedly during a 24 h period, the ratio of CD177^+/−^ neutrophils did not change (Figure 1c). This ruled out an influence of CD177 expression on the circulating levels of granulocytes, which are known to show circadian fluctuations [17]. Furthermore, in a model of oral‐cavity irritation [14] we found no difference in the ratio of CD177^+^/CD177^−^ compared to the conditions seen in the peripheral blood of the same volunteer (Figure 1d). Hence, CD177‐expression does not provide an advantage for blood extravasation to neutrophils. While the circulating numbers of CD177^+^ and CD177^−^ were stable in healthy volunteers (Figure 1b), recent studies instead suggested, that their blood‐frequency changes under conditions of sterile inflammation [8, 9]. However, in a cohort of stroke‐patients we found minimal changes over 90 days, only three individuals (9%) had a maximum change (defined as the difference between the overall highest and lowest measured CD177 value per individuum) of more than 4‐times that of the median (median_change_ = 4.3%). The total ratio of CD177^−^ to CD177^+^ cells ranged from ∼30% to near 100% and also included three CD177‐deficient (CD177^null^) individuals (Figure 1e).

### 3.2 CD177^−^ Neutrophils Do Not Express CD177 Upon Activation

**FIGURE 2:**
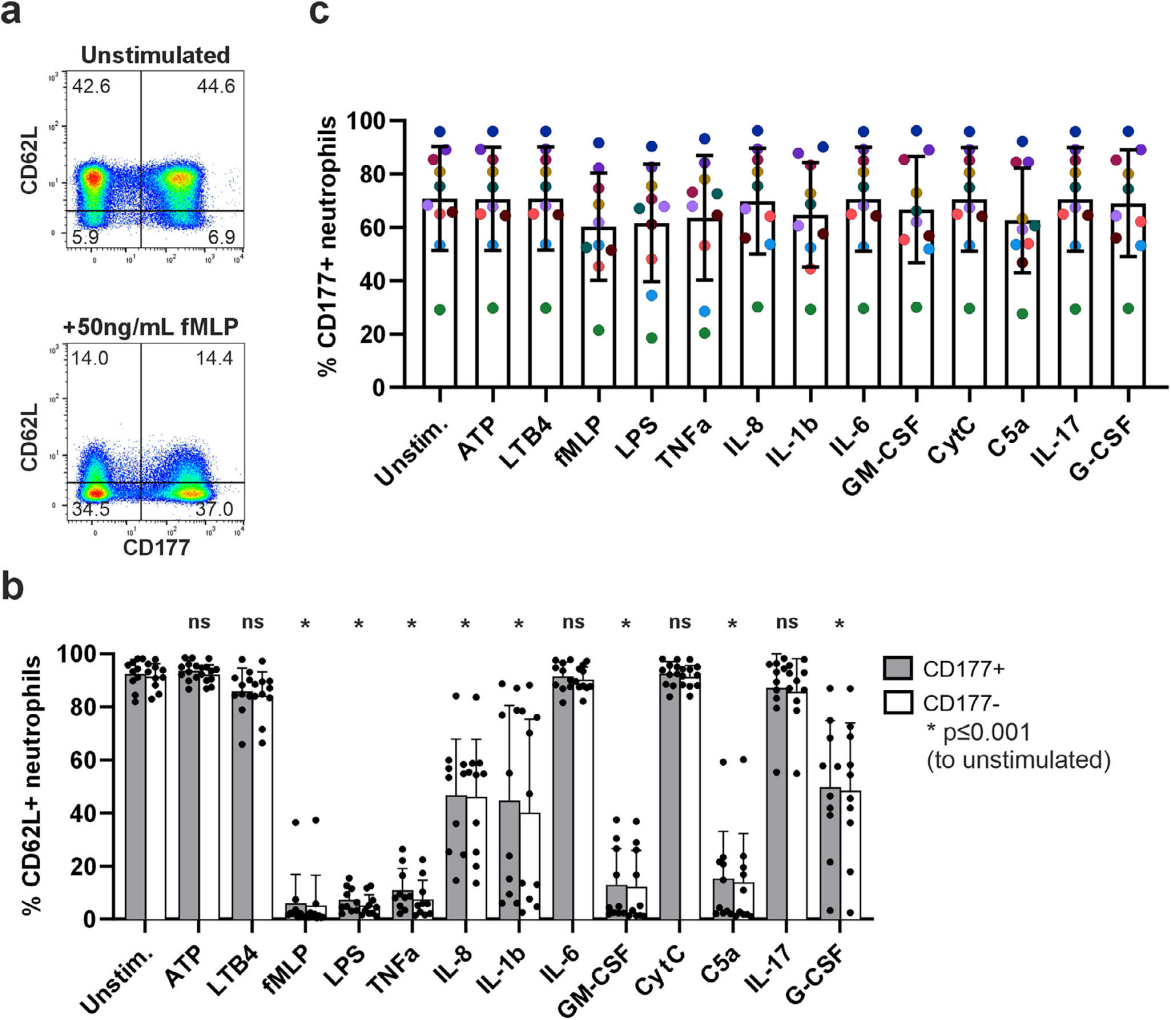
CD177 status remains unchanged upon neutrophil activation. (a) Exemplary flow cytometry plots comparing CD177 and CD62L expression patterns on neutrophils of one individual before and after stimulation with 50 ng/mL fMLP for 1 h. (b) Percentage of CD62L expression on neutrophils upon triggering with indicated stimuli for 1 h, analyzed by CD177 surface expression, data presented as mean±SD and individual values, n = 10. Compared to unstimulated all but ATP, LTB4, IL‐6, CytC, & IL 17 significantly reduced CD62L expression on the cell‐surface (* = p ≤ 0.0001). (c) Proportion of CD177^+^ neutrophils out of total neutrophil count from healthy individuals upon 1 h stimulation with the indicated stimuli, data presented as mean±SD with individual values color‐coded by donor (n = 10).

CD177 is considered an activation marker in neutrophils [18]. Hence, we tested, whether in vitro stimulation would induce CD177^−^ neutrophils to become CD177^+^. The bacterial protein‐derived tripeptide fMet‐Leu‐Phe (fMLP) is known to rapidly induce neutrophil activation via recognition through a specific fMLP‐receptor [19]. Consequently, the stimulation with fMLP led CD177^+^ neutrophils to express higher CD177‐surface levels. However, importantly, despite effective downregulation of L‐selectin (CD62L) as a sign of activation, CD177^−^ cells of the same host remained negative within the same sample (Figure 2a). Exposing cells from 10 individuals to multiple activation triggers further confirmed this observation. CD62L loss was consistent among CD177^+^ and CD177^−^ cells of the same individuals (Figure 2b), yet despite this efficient cellular activation the percentage of CD177^+^ neutrophils was not changed (Figure 2c). Although we could confirm increases of CD177‐MFI on CD177^+^ neutrophils when challenged with strong triggers (Figure S1a), CD177^−^ cells always remained negative. Donor‐resolved trajectories reveal that a subset of individuals shows a reduction in the CD177^+^ fraction under strong activation conditions, most prominently with LPS (Figure 2C). This decrease is restricted to the CD177^+^ population and does not lead to emergence of CD177 expression in previously CD177^−^ neutrophils. Given that CD177 is a GPI‐anchored protein capable of being shed from the cell surface [20], we believe this might be a stimulus‐induced surface loss of CD177 in responsive donors rather than phenotypic conversion.

**FIGURE 3:**
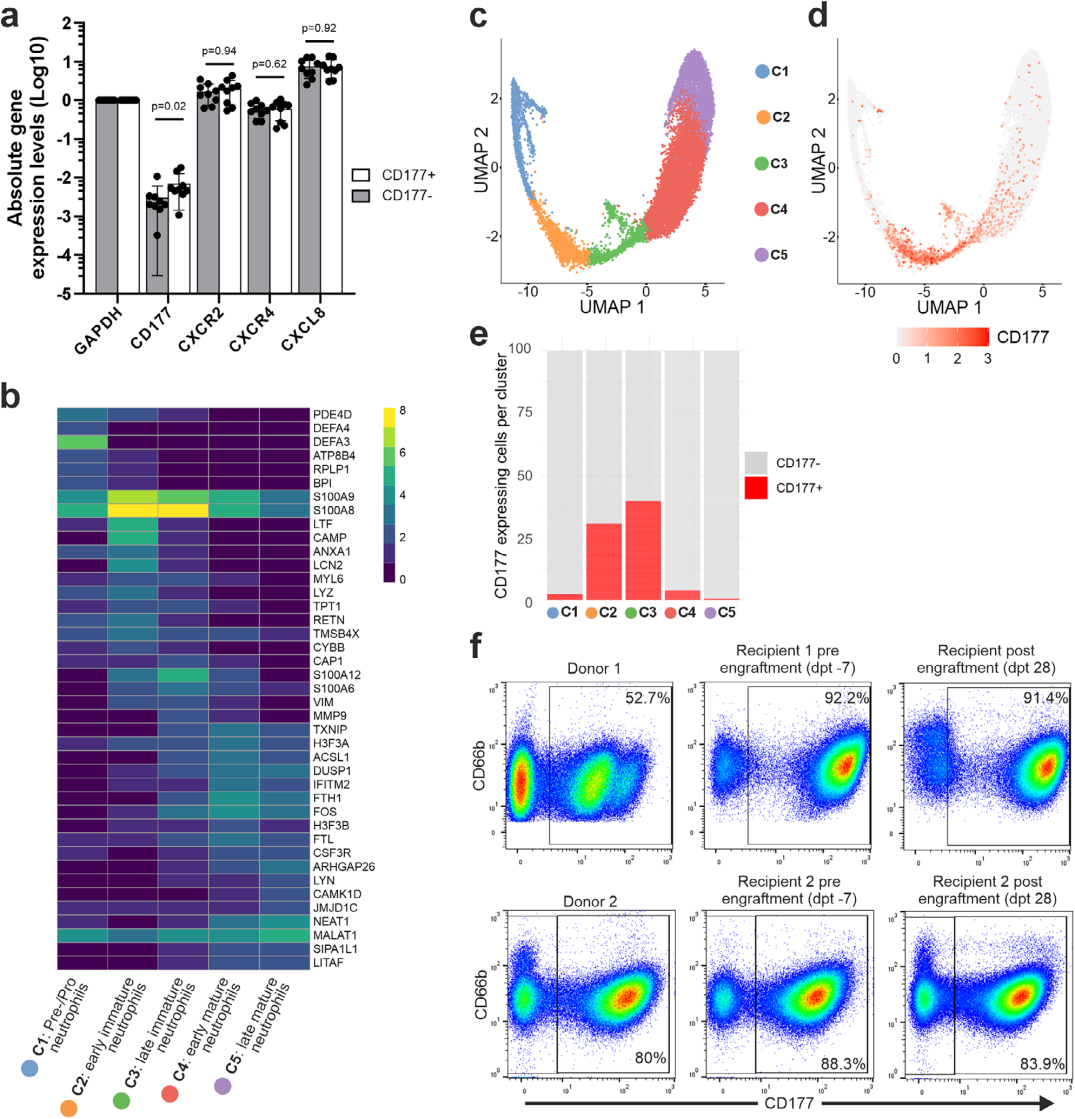
CD177 mRNA expression occurs in immature neutrophils. (a) Absolute transcript numbers of CD177 and established neutrophil markers CXCR2, CXCR4, and CXCL8 normalized to GAPDH, in CD177 sorted neutrophils. Values given as mean±SD, including individual values; (*: p ≤ 0.05), n = 9. (b) Gene signatures (mRNA expression) from different clusters of bone marrow neutrophils, following the patterns of maturation state, calculated from single‐cell RNA sequencing data as described in Montaldo et al. 2022 (18). (c) Single‐cell RNA sequencing of bone marrow neutrophils and their precursors, UMAP and clustering were calculated via unbiased highly variable genes and kmers, respectively (d) Corresponding expression of CD177 mRNA in individual cells within identified developmental neutrophil stages, n = 12 924. (e) Quantification of the amount of CD177^+^ expressing vs. CD177^−^ cells in the identified developmental stages shown in b and c. (f) Flow cytometric analysis of the CD177 expression pattern on neutrophils of two patients with acute lymphoblastic leukemia 7 days before (dpt ‐7) (middle panel) and 28 days after (dpt 28) (right panel) hematopoietic stem cell transplantation. The CD177 expression pattern of the respective donor is shown in the left panel for comparison. The patients showed a clinically confirmed donor chimerism of >99% on dpt 28.

Interestingly, the differences in CD177 protein expression between the two subsets were not reflected at the mRNA level in mature blood neutrophils. In both, CD177^+^ and CD177^−^ neutrophils, we found substantially lower amounts of CD177 mRNA compared to mRNA‐levels of the housekeeping gene GAPDH or the more neutrophil specific genes CXCR2, CXCR4 or CXCL8 (Figure 3a). This apparent mRNA‐protein dissociation, however, is consistent with a prevalent feature of human neutrophils, where transcriptomic and proteomic profiles are broadly decoupled throughout myeloid differentiation [21]. The phenomenon reflects the limited transcriptional activity of mature neutrophils, which rely heavily on post‐transcriptional regulation and pre‐existing protein stores rather than de novo mRNA synthesis. Given this characteristic decoupling in mature cells, we sought to determine when CD177 expression is established during neutrophil development, prompting us to examine immature bone marrow precursors. Consistent with earlier bulk mRNA‐sequencing [22], a re‐exploration of more recently published single‐cell‐RNA‐sequencing data of human neutrophils and their precursors from bone marrow samples [13] showed that CD177 mRNA is produced mainly by early and late immature bone‐marrow neutrophils (Figure 3b–d) in fractions similar to what is later found in peripheral blood (Figure 3e). Hence, CD177 mRNA/protein production peaks during early neutrophil differentiation and is largely terminated in mature cells. The slight increase in CD177 surface‐expression seen after stimulation (Figure S1a) is therefore likely recruited from pre‐formed intracellular stores as shown by differential surface‐ and intracellular CD177‐labelling followed by analysis via flow cytometry (Figure S1b).

### 3.3 The Hematopoietic Niche Controls CD177 Expression

The analysis of single cell data (Figure 3c–e) suggested that the CD177 phenotype of mature neutrophils is predefined in precursors and should hence be transplantable through stem cells. A patient with acute lymphoblastic leukemia (ALL) scheduled for allogeneic stem cell transplantation allowed to test this assumption. We investigated the CD177 status of the stem cell donor, and the patient before and 28 days after transplantation, when donor chimerism was >99.7%. Unexpectedly, the CD177 expression pattern on circulating neutrophils post engraftment corresponded precisely to the pretransplant pattern and did not change to the donor state (Figure 3f, upper panel). We were able to confirm this observation in a second donor and the corresponding recipient, who suffered from ALL, although the phenotype was less obvious here since the donor and recipient were much more similar in their initial CD177 expression pattern (Figure 3f, lower panel, Table S1). These data show that the bone marrow microenvironment (stem cell niche) is essential for defining the CD177 expression pattern of human neutrophils, with stem cells exhibiting a highly plastic response to niche signals.

### 3.4 CD177^−^‐Neutrophils are Morphologically Indistinct from CD177+‐Cells but Show Large Molecular Differences

**FIGURE 4:**
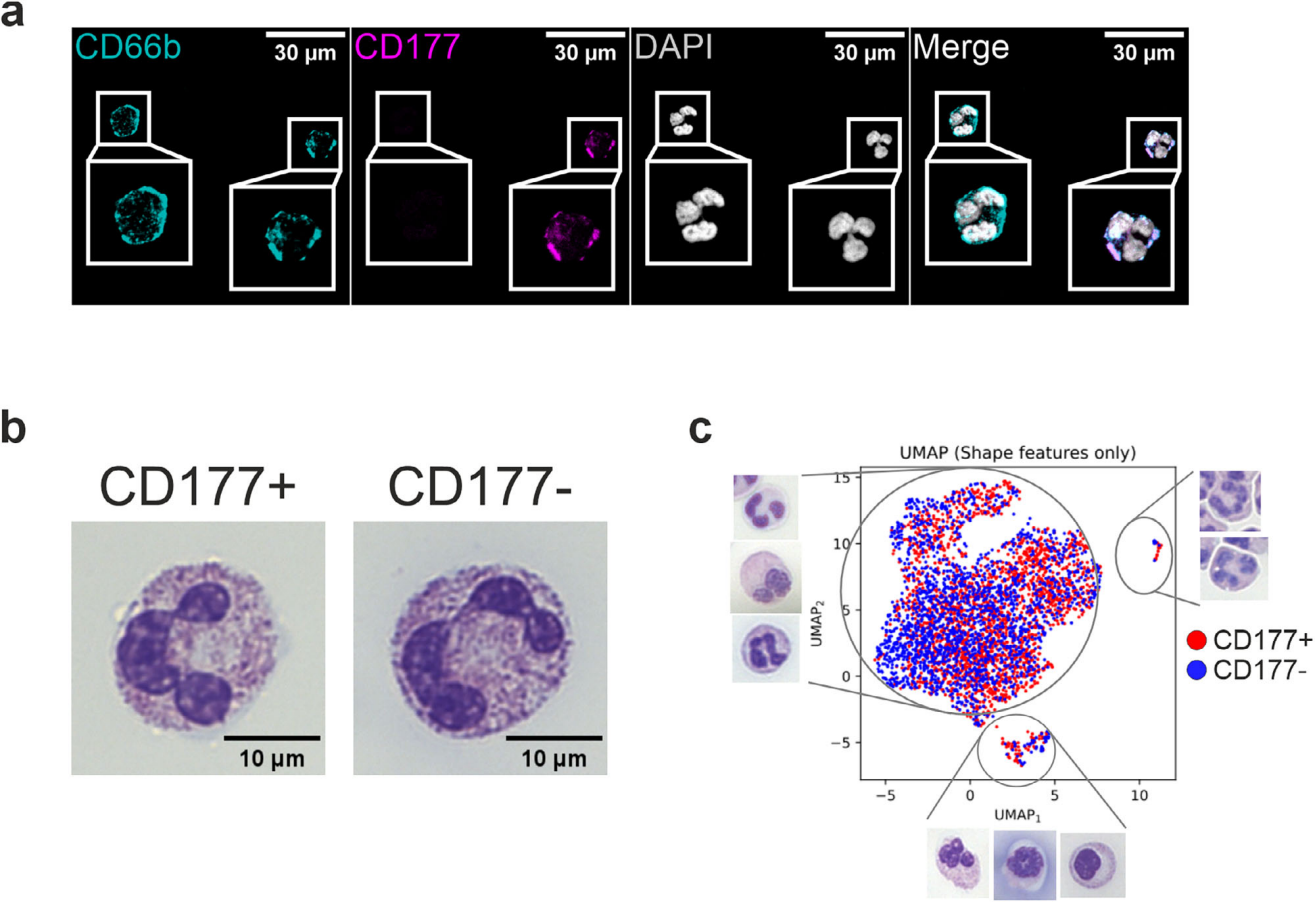
CD177 protein expression is developmentally fixed and absent from mature CD177^−^ neutrophils. (a) Confocal imaging of neutrophils isolated from peripheral blood of a healthy donor, stained for CD66b (cyan), CD177 (magenta), and DAPI (grey) as nuclear counterstain; scale bar is 30 µm. (b) May‐Grünwald‐staining of FACS‐sorted and cytospun CD177^−^ and CD177^+^ neutrophils used as input data for multidimensional feature comparison; scale bar is 10 µm. (c) UMAP dimension reduction of accessed shape features derived from >4000 sorted CD177^−^ and CD177^+^ neutrophils, including representative images of resulting clusters.

To morphologically characterize the two subsets, neutrophils were immobilized on coverslips and analyzed by multicolor confocal microscopy. This confirmed that within the same donor CD66b^+^ neutrophils could either express or lack the expression of CD177 protein, both intracellularly and at the surface (Figure 4a, Figure S1b) confirming previous findings [23]. Next, cells FACS‐sorted via CD177 surface‐expression were cytospun and May‐Grünwald‐stained (Figure 4b). Around 2000 single cell images from each subtype were analyzed computationally applying >80 morphological features (Figure S2a/b). However, dimension‐reduction of these features revealed no separation between the two initially sorted populations (Figure 4c). Additional statistical tests revealed that CD177^+/−^ cells only differed slightly in size (Figure S2c). Sorted CD177^−^ neutrophils showed significant survival advantages in vitro (Figure S3a). However, in other functional aspects including ROS production (Figure S3b) and bacterial phagocytosis in vitro (Figure S3c/d) CD177^+^ cells resembled CD177^−^ neutrophils. Collectively these data suggested, that CD177^−^ cells represent a stable neutrophil subtype that remains CD177^−^ under stimulation but appears to be indistinguishable from CD177^+^ neutrophils in classical measures of neutrophil activity.

**FIGURE 5:**
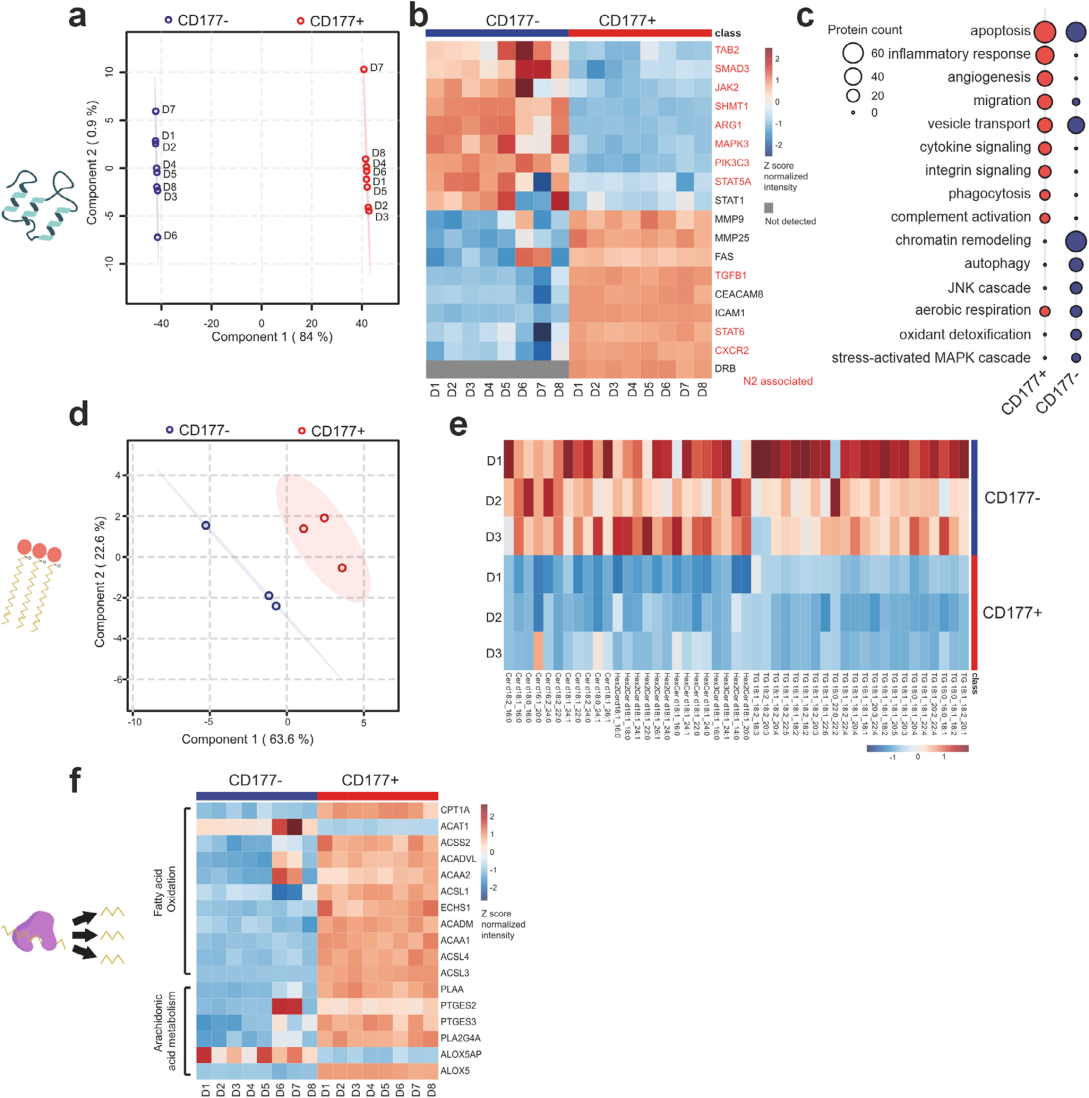
Integrated proteomic‐lipidomic profiling reveals distinct molecular signatures in CD177‐defined neutrophils. (a) PCA analysis of whole proteomics data of FACS‐sorted CD177^−^ and CD177^+^ neutrophils from 8 healthy donors (labeled D1‐D8). (b) Heatmap showing expression patterns of N1(black)/N2(red)‐associated proteins found in FACS‐sorted CD177^−^ and CD177^+^ neutrophils from 8 healthy donors (D1‐D8). (c) REACTOME pathway analysis of neutrophil proteomes of sorted CD177^−^ and CD177^+^neutrophils (n = 8). (d) PCA analysis of whole lipidomic data of MACS‐sorted CD177^−^ and CD177^+^ neutrophils from 3 healthy donors. (e) Heatmap showing lipids found in MACS‐sorted CD177^−^ and CD177^+^ neutrophils from 3 healthy donors. (f) Heatmap depicting expression patterns of proteins associated with fatty acid oxidation (top) and arachidonic acid metabolism (bottom) in FACS‐sorted CD177^−^ and CD177^+^ neutrophils from 8 healthy donors (D1‐D8).

This similarity between CD177^−^ and CD177^+^ cells made it hard to explain their large functional differences seen in various diseases [3, 8, 9]. Hence, we subjected sorted CD177^−^ and CD177^+^ neutrophils of eight donors to proteomic profiling as described [14]. This revealed profound molecular distinctions between the two subtypes. In fact, CD177^+^ and CD177^−^ cells of the same donor were molecularly more distinct than all CD177^+^ or CD177^−^ cells from all donors (Figure 5a/b). Interestingly, CD177^−^ cells over‐expressed many proteins previously associated with pro‐tumorigenic neutrophils, which are also termed N2‐neutrophils [24] (Figure 5b). We have shown earlier, that pro‐tumorigenic neutrophils infiltrating human head‐and‐neck cancer (HNC) are characterized by the high expression of arginase 1 and in their physical neighborhood T cells are less active and do not proliferate [25]. Thereby, neutrophil‐derived arginase 1 has been shown to directly suppress T cell activation [26]. Fitting to a potential pro‐tumorigenic role of CD177^−^ neutrophils, they consistently expressed about 3.5‐fold more arginase 1 protein than their CD177^+^ counterparts in the same donors (Figure 5b). Additionally, it has been shown previously that arginase 2, which is active in mitochondria of cancer cells, induces the overexpression of mitochondrial serine hydroxymethyltransferase (SHMT2) [27]. We hypothesized, therefore, that similar mechanisms might be active in the cytoplasm of neutrophils. Hence, we measured the amount of cytoplasmic SHMT1 protein and found it to be about 4.5‐fold upregulated in CD177^−^ compared to CD177^+^ neutrophils (Figure 5b). This provided an independent measure that circulating CD177^−^ neutrophils are equipped with a much higher arginase 1 activity than their CD177^+^ counterparts. In our previous study we had also shown that a population of neutrophils expressing HLA‐DR molecules in HNC tissue is closely positioned to T cells showing signs of activation and proliferation in situ [25]. Interestingly, we could not detect any HLA‐DR protein in CD177^−^, while CD177^+^ neutrophils expressed HLA‐DRB robustly (Figure 5b). Further, REACTOME analyses of the proteomics‐data revealed functional differences related to inflammation, antioxidant activity and chromatin remodeling between the two subsets (Figure 5c). Overall, these data show that despite their close similarity in many traditional measures, CD177^−^ cells differ greatly from CD177^+^ neutrophils when analyzed using deep molecular profiling.

Next, we subjected sorted CD177^+^ and CD177^−^ neutrophils of the same donors to comprehensive analysis of their lipid composition using mass spectrometry‐based lipidomics profiling (Figure 5d,e). This showed that, again, CD177^−^ cells strongly differed from CD177^+^ cells (Figure 5d). Specifically, CD177^+^ contained much lower levels of triacylglycerides and sphingolipids than CD177^−^ cells (Figure 5e). The breakdown of triacylglycerides is a prominent source for fatty acid ß‐oxidation or arachidonic acid release [28, 29, 30], whereas sphingolipids have been documented to be potent for modulating immune cells [31]. Hence, we investigated, whether the proteomic dataset would provide hints for the increased activity of fatty acid catabolism and altered sphingolipid abundance. Indeed, CD177^+^ cells were highly enriched in a large number of proteins associated with fatty acid breakdown and arachidonic acid metabolism (Figure 5f), which further supported the lipidomics analyses. In summary, CD177^+^ neutrophils appear to have a more increased fatty acid breakdown machinery running or use more of their lipid‐mediator precursor pool (sphingolipids, arachidonic acid) compared to CD177^−^ cells in the same host.

### 3.5 CD177^−^‐Neutrophils are Preferentially Recruited Into HNC Tumor Tissue and Support Tumor Growth

**FIGURE 6:**
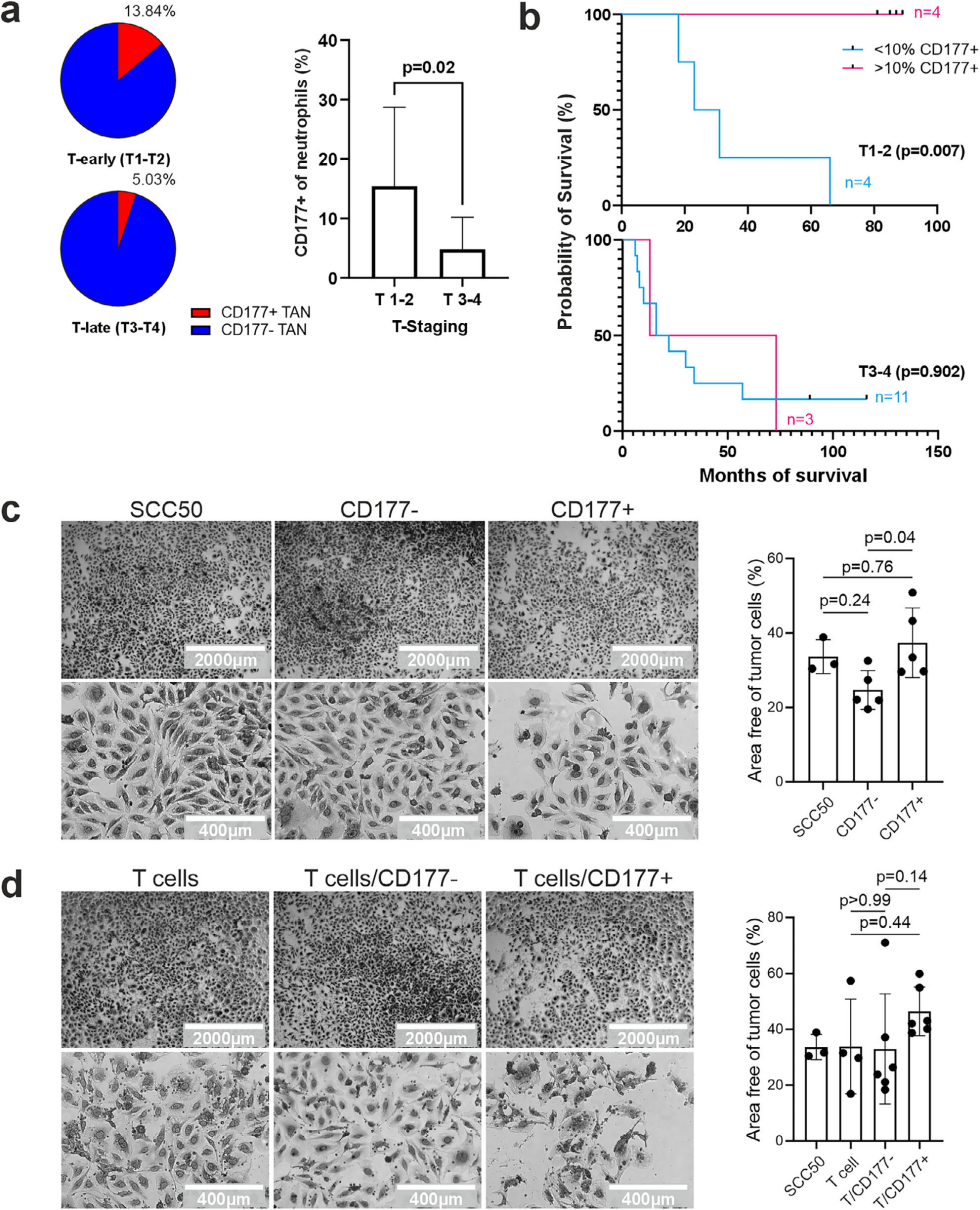
CD177 delineates an immunoregulatory neutrophil subtype in head‐and‐neck cancer. (a) Proportion of CD177 expressing tumor‐associated neutrophils (TAN) in head‐and‐neck cancer (HNC) tissue separated by tumor progression stage (early T1/2, late T3/4), data given as mean+SD (* = p ≤ 0.05), n(early) = 9; n(late) = 17. (b) Kaplan‐Meier curves, showing long‐term survival of HNC patients with >10% CD177^+^ TANs found within the tumor stroma (magenta) against patients with <10% CD177^+^ (blue) separated by early stages (T1/T2; top) and late stages (T3/T4; bottom). Significant differences according to the Log‐rank test were found for early‐stage tumors. (c) Microscopy images of UT‐SCC‐50 cells cocultured with the indicated neutrophil subpopulation for 48 h and quantification of cell‐free area. (d) Microscopy images of UT‐SCC‐50 cells cocultured with T cells and indicated autologous CD177‐FACS‐sorted neutrophils for 48 h and quantification of cell free area.

Our proteomic analyses strongly linked CD177^−^ cells to a pro‐tumorigenic [32] N2‐like phenotype (Figure 5a–c). Hence, we tested, whether human HNC, which is strongly influenced by tumor‐associated neutrophils (TANs) [25], would preferentially recruit CD177^−^ cells. Indeed, histological analyses of tumor‐tissue‐micro‐arrays covering all stages of HNC from 26 patients showed a selective enrichment of CD177^−^ TANs, especially toward late stage tumors (T3/T4, Figure 6a and Figure S5a), while levels of CD177^−^ and CD177^+^ neutrophils in the blood of the same patients were not significantly different (Figure S5). Most notably, longitudinal follow‐up analyses revealed that, in early‐stage disease (T1/2), lower intratumoral infiltration by CD177^−^ neutrophils was associated with significantly improved patient survival. In contrast, at more advanced stages, the abundance of CD177^+^ neutrophils within tumor tissue did not correlate with overall survival (Figure 6b).

Finally, we tested the direct influence of both neutrophil subtypes sorted from healthy donors on the growth of squamous‐cell carcinoma cell‐lines in vitro. This identified two important functional differences between the two neutrophil subtypes. First, in the absence of tumor‐killing T cells, sorted CD177^+^ neutrophils significantly impaired tumor cell growth, while CD177^−^ of the same donor even slightly enhanced the growth of the cell line in vitro (Figure 6c). Second, when co‐incubated with potentially tumor‐lytic T cells of the same donor, CD177^+^ neutrophils enhanced their killing capacity, while CD177^−^ neutrophils lacked this ability (Figure 6d). This provided a rationale for the effects of CD177^+^ and CD177^−^ neutrophils on the course of HNC in human patients seen before (Figure 6b). In summary these data showed that HNC preferentially recruit CD177^−^ pro‐tumoral neutrophils from the circulation that promote tumor progression.

## 4 Conclusions

Although already described in 1992 [33], the bimodal CD177 expression pattern on human neutrophils is still under‐evaluated. Its prevalent description in the literature as a mere maturation marker contributes to the common misconception [11, 18]. Here we show that CD177 expression or its lack in fact delineates stable subpopulations of circulating neutrophils under homeostatic conditions and upon stimulation. This stability raises the question whether these subsets serve distinct functional roles.

While previous investigations into the role of CD177^+^ cells often led to inconsistent results regarding their immune functions [9, 34, 35], the specific role of CD177^−^ cells was so far largely ignored. Since CD177^null^ individuals exist without obvious survival disadvantages [4], a potential difference between the two subpopulations was considered irrelevant. We show that this assumption was premature. Whether individuals who completely lack CD177 expression carry an elevated cancer risk remains an important clinical question that, to the best of our knowledge, has not yet been addressed in a population‐based study.

Our best explanation for the existence of CD177^−^ neutrophils is a functional diversification, with non‐expressing cells serving as a more immunoregulatory subset, potentially to offset heightened functions of CD177^+^ cells [1, 5]. Supporting this, our molecular characterization shows that CD177^−^ neutrophils exhibit an N2 pro‐tumoral phenotype and do not stimulate T cell tumor killing activity, while CD177^+^ cells do. Although often described as pro‐tumoral, N2‐polarized neutrophils also show strong anti‐inflammatory effects and are less cytotoxic [36, 37]. Furthermore, the association with patient survival observed here, delineates TAN‐CD177 as a potential, future biomarker for early tumor‐risk‐assessment.

In the light of excessive literature on myeloid‐derived suppressor cells (MDSC), it is also tempting to speculate that CD177^−^ neutrophils might be one type of MDSC. The most recent literature mentions high expression of arginase 1 and tumor supportive activity as defining features [38, 39, 40]. Our data show that these features are both present in CD177^−^ neutrophils. A conceptually related neutrophil phenotype was described by Marini et al., who showed that mature CD10^+^ and immature CD10^−^ neutrophils display opposite immunoregulatory effects on T cells, with CD10^+^ cells suppressing T‐cell proliferation also in an arginase 1 dependent manner [41]. This parallels our findings regarding the immunosuppressive role of CD177^−^ cells, although key differences remain. CD10 serves as both a maturation and activation marker on neutrophils, with its expression positively correlating with other activation markers such as CD11b and CD66b [42, 43]. While CD10^+^ neutrophils are predominantly mature cells and CD10^−^ neutrophils include immature forms that emerge under inflammatory conditions. In contrast, the two distinct CD177 subpopulations we describe are present in every healthy individual (except full knockouts), persist independent of inflammatory contexts, and consist of neutrophils of identical maturation states, indicating that CD177 defines functionally distinct neutrophil lineages rather than activation or maturation states.

Regarding the molecular basis of this immunosuppressive program, lipid species accumulating in CD177^−^ neutrophils may play an important role. Sphingolipids in particular are established regulators of immune cell survival, inflammatory signaling and autophagy [31], potentially contributing to the prolonged survival we noted for this subset. Conversely, the observed upregulation of β‐oxidation components in CD177^+^ cells provides a mechanistic basis for the relative depletion of triglycerides in CD177^+^ neutrophils. Interestingly, lipid driven metabolic reprogramming was recently identified as a way by which the TME shapes neutrophils toward an immunosuppressive, MDSC‐like phenotype, via a process in which fatty acid uptake and lipid accumulation features prominently [44]. Future molecular analyses must hence define how these functionally distinct cells can be selectively targeted, a significant problem in present day cancer immunotherapy [39].

We also provide mechanistic insights into CD177 expression‐regulation. While monoallelic silencing via CpG‐methylation is known as the main mechanism underlying bimodality, the specific factors driving it have not been investigated in depth [5]. We show that early and late immature neutrophils in the bone marrow are the likely cells in which the decision to express CD177 is made. Our observations in human stem cell transplantation suggest that CD177‐silencing or ‐activation is mediated by distinct bone marrow niches, which are resistant to the harsh conditioning regimen used in human stem cell transplantation to treat acute lymphoblastic leukemia. Independent of the epigenetic patterns of their donor origin, hematopoietic stem cells show a plastic response to programming‐triggers of the local bone‐marrow microenvironment. Inflammation‐induced changes in this niche might explain why increases in CD177^+^ cells are detectable under certain conditions such as after kidney transplantation [23] or 6–72 h post‐stroke [9], although in our stroke cohort, no durable changes were observed over 3 months. Further research needs to elucidate the cellular source and molecular identity of niche factors controlling CD177 expression in human neutrophil progenitors.

In this context, the question of what CD177 itself contributes mechanistically to the observed functional differences of neutrophils also deserves consideration. As a GPI‐anchored protein, CD177 lacks an intracellular domain and cannot signal autonomously. However, GPI‐anchored proteins are well known to reside in lipid raft nanodomains that spatially co‐localize with integrin nanoclusters and serve as nucleation sites for receptor crosstalk and cell adhesion signaling [45]. Consistent with this, Mac‐1 (CD11b/CD18) has been identified as a transmembrane adaptor for CD177 on the neutrophil surface [46] and antibody‐mediated ligation of CD177 was shown to promote β2 integrin activation and downstream signaling in the context of neutrophil migration [1]. Whether such co‐receptor activity is engaged by a physiological ligand in vivo, for example within the tumor microenvironment, has not been demonstrated. At the present state of knowledge, we therefore regard CD177 expression primarily as a stable surrogate marker of transcriptionally distinct neutrophil subtypes, while its potential functional contributions as a co‐receptor require dedicated future investigations.

Beyond questions of expression regulation, our data demonstrating the large functional difference of CD177^+^ and CD177^−^ cells in the same host require research into methods to selectively target each subset. For example, recent approaches to make neutrophils “visible” for magnetic resonance imaging in vivo using CD177‐targeted tracers [47] now must be viewed as problematic, as they will overlook a potentially large part of the neutrophil pool in individuals. Ideally it would be possible to define traceable markers for both subtypes individually to then be able to identify each subtype selectively in relevant tissues. Finally, what remains to be defined is a context in which CD177^−^ cells are overtly beneficial. It is implausible that a robust epigenetic system maintaining CD177 silence in a subset of neutrophil progenitors has been conserved through evolution without conferring a survival advantage.

While our data provide multiple lines of evidence for functional dichotomy between CD177 subsets, certain methodological aspects deserve careful consideration. Given the multi‐center, multi‐modal design of this study, several factors should be considered when interpreting the results. First, neutrophils from different cohorts were processed using distinct isolation strategies (MACS‐based negative selection for most mechanistic experiments, whole blood staining in the stroke cohort, density gradient isolation in some HNC‐related experiments), and proteomic and lipidomic analysis relied on FACS‐ and MACS‐sorted subsets, respectively. Although these workflows were chosen deliberately to balance purity, processing time and biochemical stability, they may influence absolute levels of selected readouts and complicate direct cross‐comparison of effect sizes across assays and cohorts. Second, flow cytometry data were acquired on different instruments and analyzed in different software environments, which in principle can introduce subtle differences in gating near threshold regions. To mitigate these issues, all key mechanistical comparisons were performed within internally consistent pipelines in a single center, using standardized operating procedures. Additionally, all flow cytometry datasets were manually gated and re‐checked by the same experienced operator. Third, some of the most informative datasets are based on modest sample sizes, including the stem cell transplantation series (n = 2 donor‐recipient pairs) and the tumor‐killing assay (n = 4 donors), so these findings should be viewed as proof‐of‐concept for the underlying mechanisms rather than definitive population estimates. Fourth, our in vitro culture conditions (standard media, atmospheric oxygen, absence of autologous plasma) differ from the physiological environment in which neutrophils circulate in vivo. Neutrophils cultured under such conditions experience hyperoxic stress that accelerates apoptosis compared to in vivo circulation, where cells are maintained in autologous plasma under lower oxygen tension [48, 49]. The rapid viability decline observed in our time‐course experiments (Figure S4a) is consistent with published reports of neutrophil fragility under standard culture conditions, limiting assessment of phenotype stability and functional differences to approximately 8–10 h. Nevertheless, the differential survival between CD177^+^ and CD177^−^ subsets was evident within the viable timeframe, and the functional dichotomy was confirmed across multiple independent short‐term assays. Although the exclusive focus on human samples and clinically relevant cohorts is a major strength of this work, it inevitably restricts the range of experimental perturbations and longitudinal interventions that can be performed. Hence, our conclusions largely rely on ex vivo assays and observational associations rather than interventional in vivo studies. Finally, CD177 in our study functions as a stable surrogate marker for deeply imprinted neutrophil programs and we do not delineate a direct molecular effector function of the CD177 protein itself. Together, these limitations should be considered when extrapolating our findings to other cohorts, disease entities or experimental contexts.

Taken together, our findings establish CD177 expression as a stable, epigenetically imprinted marker of functionally distinct human neutrophil subsets and identify CD177^−^ cells as a durable tumor‐supportive, immunomodulatory population that must be considered in future diagnostic, prognostic, and therapeutic strategies.

## Author Contributions

M.J., A.Be., M.G., D.M.B., J.J. designed experiments, M.J., A.Be. contributed in equal parts. M.J., A.Be., S.G., E.P., B.A., D.N., K.S., L.K., E.H., M.K., N.K., A.Ba., M.R. performed experiments and primary data acquisition. T.T., Y.Z., B.F. N.B.L., T.S., J.G., N.H., A.M.Y., M.K., J.J. provided additional data on patient samples. S.Lo., K.B., A.K. assisted in donor blood collection. H.W., J.C., D.N. provided customized computational data analysis. R.S. suggested and F.Z. analyzed RNA‐seq data. M.J., A.Be., M.G. wrote the original manuscript with the help of B.G., S.La., A.G., S.H., J.J., A.H., O.S., H.C.R., A.S. and D.M.H. All authors reviewed the manuscript prior to submission.

## Conflicts of Interest

H.C.R. received consulting and lecture fees from Abbvie, Roche, KinSea, Vitis, Cerus, Lilly, Novartis, Takeda, AstraZeneca, Vertex, and Merck. He also received research funding from AstraZeneca and Gilead Pharmaceuticals. He is a co‐founder of CDL Therapeutics GmbH. The other authors declared no conflicts of interest.

## Supporting information




**Supporting File**: advs76236‐sup‐0001‐SuppMat.docx.

## Data Availability

The mass spectrometry proteomics data have been deposited to the ProteomeXchange Consortium via the PRIDE [50] partner repository with the dataset identifier PXD068607 (Username: reviewer_pxd068607@ebi.ac.uk, Password: zeeFuPY6t2QE). Lipidomics data have been deposited to the data repository Zenodo under the dataset identifier https://doi.org/10.5281/zenodo.18785694. All data are publicly available as of the date of publication. Additional information required to reanalyze the data will be shared by lead contact upon request. An executable version of the used ADAPT script for detection of viable neutrophils, with detailed documentation can be accessed at: https://github.com/Deuto94/ADAPT.
