# Supplementary material for: CD177 Deficiency Defines a Stable Subtype of Human Neutrophil Granulocytes with Tumor Promoting Activity
Source: Adv Sci (Weinh). 2026 Jun 22;13(52):e76236. doi: 10.1002/advs.76236 (PMC13336833; doi:10.1002/advs.76236)
Supplement: Supplementary file 1 [file ADVS-13-e76236-s001.docx]

**Supporting Information for**

**CD177 deficiency defines a stable subtype of human neutrophil granulocytes with tumor promoting activity**

Marcel Jung^1#^, Alexander Beer^1#^, Susmita Ghosh^2^, Ekaterina Pylaeva^3^, Belal Alshaar^2^, Tobias Tertel^4^, Nils Bastian Leimkühler^5^, Thomas Schroeder^5^, Janine Gronewold^6^, Nina Hagemann^6^, Ayan Mohamud Yusuf^6^, Benedikt Frank^6^, Yiqiao Zhang^6^, Dennis Nagel^1^, Kim Schloeßer^2^, Laura Karsch^1^, Emily Hedtfeld^1^, Sabrina Lohmann^1^, Kathrin Blank^1^, Andreas Kraus^1^, Max Krumbein^3^, Nastassia Kabankova^3^, Hongxiao Wang^2,7^, Almke Bader^8^, Mathis Richter^9^, Fengjun Zhang^9^, Bernd Giebel^4^, Stephan Lang^3,10^, Anika Grüneboom^2^, Daniela Maier-Begandt^8^, Anja Hasenberg^1^, Oliver Soehnlein^9^, Hans Christian Reinhardt^5,11,12^, Sven Heiles^2,13^, Jianxu Chen^2^, Jadwiga Jablonska^3,10^, Albert Sickmann^2,14^, Dirk M. Hermann^6^, Matthias Gunzer^1,2^

Lead contact: Matthias Gunzer

Email: matthias.gunzer@uk-essen.de

**This PDF file includes:**

Table S1

Figures S1 to S5

Figures

**Fig. S1.** **Flow cytometry analysis of CD177 surface mobilization. a** Mean fluorescent intensity (MFI) of CD177 signal detectable on isolated neutrophils upon triggering with varying stimuli, measured on the same set of data as Fig. 2b/c, data represented as mean±SD plus individual values (*=p≤0.05), n=10. **b** Neutrophils from three donors (D1, D8 & D11) were surface-stained for CD66b and CD177 before intracellular staining with the same CD177 antibody coupled to a different fluorophore. All cells were gated for singlets and neutrophils (CD66b^+^). The majority of CD177 expressing neutrophils appears double positive for extra- and intracellular CD177. In contrast, the cells being CD177^-^ on the cell surface can also not be stained intracellularly, hence showing the complete absence of CD177 protein in these cells.

Table S1. Flow cytometric parameters of CD177 subpopulations in bone marrow donor-recipient pairs. Shown are the mean fluorescent intensities (MFI) of the individual subpopulation as well as the fold change in MFI between CD177^+^/CD177^-^ populations and the difference (peak-to-peak distance) between MFI values for comparison between donor and recipient expression patterns of the two donor-recipient-pairs shown in Figure 3f.

| Sample | | MFI^CD177+^ [AU] | MFI^CD177-^ [AU] | MFI-fold-change (CD177+/CD177-) | Δ MFI^CD177+^ - MFI^CD177-^ |
| --- | --- | --- | --- | --- | --- |
| **Pair 1** | Donor 1 | 23.80 | 0.27 | 88.15 | 23.53 |
|  | Recipient 1 pre | 247 | 0.7 | 352.86 | 246.30 |
|  | Recipient 1 post | 270 | 1.32 | 204.55 | 268.68 |
| **Pair 2** | Donor 2 | 117 | 0.79 | 148.10 | 116.21 |
|  | Recipient 2 pre | 83.90 | 0.93 | 90.22 | 82.97 |
|  | Recipient 2 post | 103 | 1.29 | 79.84 | 101.71 |

**Fig. S2.** **Assessment of morphological features from sorted neutrophils based on CD177 expression.** **a** Workflow of artificial intelligence-driven segmentation and determination of 80 feature parameters of >4,000 high resolution single cell images. **b** Dimensional reduction of derived features via clustering algorithms UMAP (left) and t-SNE (right). **c** Statistical analysis of assessed features. Shown are the distribution plots of the three features with the highest effect size, cell size (left) and nucleus-to-cytoplasm ratio (middle) were found to be of significant difference between CD177^-^ (red) and CD177^+^ (blue) cells; p-values give the significance between group means, r‑values show the respective effect size.

**Fig. S3.** **Neutrophil functional analysis in relation to CD177 expression. a** Survival (number of living cells) over 10 h of freshly isolated neutrophils (mixed), MACS CD177^+^‑enriched (CD177^+^) or CD177^+^‑depleted (CD177-) subpopulations measured by CytoCalcein Violet 450 inclusion; data represented as mean±SD, b-values give the respective slope of the linear regression model used, multiple comparison between linear regression models revealed significant differences in slope (*=p≤0.05, ***= p≤0.001), n= 6. **b** Median fluorescence intensity (MFI) of DHR-123 as a measure of reactive oxygen species (ROS) production in neutrophils with or without prior stimulation, using 50 ng/mL PMA. MFI was measured via flow cytometry, neutrophil CD177 expression was determined via anti-CD177 surface staining, data presented as mean±SD and individual values; n=10. **c** Phagocytic uptake of E2-Crimson expressing E.coli by neutrophils. Percentage of neutrophils positive for E2-Crimson as a measure of phagocytosing cells, separated by CD177 surface expression via flow cytometry, data shown as mean±SD and individual values , n=11. **d** MFI of E2-Crimson signal in the same cells as in c, representative for the amount of phagocytosed bacteria. Values are expressed as mean±SD and individual values; n=11.

**Fig. S4.** **Differentially abundant lipids in CD177‑defined neutrophils. a** Volcano plot showing differentially abundant lipids in CD177^+^ vs CD177^-^ neutrophils (Log2 fold change ≥ ±1, p-value <0.05), color-coding indicates lipid species: triglycerides (TG, red), phosphatidylserine (PS, blue), hexosylceramides (HexCer, green) and phosphatidylethanolamine (PE, yellow).

**Fig. S5.** **Recruitment of CD177- neutrophils in head and neck cancer tumor tissue. a** Representative immunofluorescence staining of tumor tissue sections of a patient with high CD177^+^ neutrophil count (left) against a low CD177^+^ patient (right). Samples were stained for CD177 (red), CD66b (green) and DAPI (blue); scale bar is 50µm. **b** Comparison of neutrophil composition found within tumor tissue of different tumor progression stages to healthy donor and patient blood proportions. HD n=16, patients with varying tumor progression (T1 [n=2], T2 [n=2], T3 [n=2 ]). Values given as mean.
